# Stress-induced obesity in mice causes cognitive decline associated with inhibition of hippocampal neurogenesis and dysfunctional gut microbiota

**DOI:** 10.3389/fmicb.2024.1381423

**Published:** 2024-10-30

**Authors:** Yu-e Liu, Zhihuang Zhao, Haili He, Liangyuan Li, Chenghong Xiao, Tao Zhou, Zili You, Jinqiang Zhang

**Affiliations:** ^1^Guizhou University of Traditional Chinese Medicine, Guiyang, China; ^2^The Center of Psychosomatic Medicine, Sichuan Provincial Center for Mental Health, Sichuan Provincial People’s Hospital, University of Electronic Science and Technology of China, Chengdu, China

**Keywords:** stress, obesity, depression, cognitive function, gut microbiome, adult hippocampus neurogenesis, microglia

## Abstract

Effects of stress on obesity have been thoroughly studied in high-fat diet fed mice, but not in normal diet fed mice, which is important to clarify because even on a normal diet, some individuals will become obese under stress conditions. Here we compared mice that showed substantial weight gain or loss under chronic mild stress while on a normal diet; we compared the two groups in terms of cognitive function, hypothalamic–pituitary–adrenal signaling, neurogenesis and activation of microglia in hippocampus, gene expression and composition of the gut microbiome. Chronic mild stress induced diet-independent obesity in approximately 20% of animals, and it involved inflammatory responses in peripheral and central nervous system as well as hyperactivation of the hypothalamic–pituitary–adrenal signaling and of microglia in the hippocampus, which were associated with cognitive deficits and impaired hippocampal neurogenesis. It significantly increased in relative abundance at the phylum level (Firmicutes), at the family level (*Prevotellaceae ucg − 001* and *Lachnospiraceae NK4a136*), at the genus level (*Dubosiella* and *Turicibacter*) for some enteric flora, while reducing the relative abundance at the family level (*Lactobacillaceae* and *Erysipelotrichaceae*), at the genus level (*Bacteroidota, Alistipes, Alloprevotella, Bifidobacterium and Desulfovibrio*) for some enteric flora. These results suggest that stress, independently of diet, can induce obesity and cognitive decline that involve dysfunctional gut microbiota. These insights imply that mitigation of hypothalamic–pituitary–adrenal signaling and microglial activation as well as remodeling of gut microbiota may reverse stress-induced obesity and associated cognitive decline.

## Highlights

- Chronic stress can induce diet-independent obesity in mice.
- Stress-induced obesity involves alterations in the gut microbiome and hyperactivation of the hypothalamic–pituitary–adrenal axis.
- Stress-induced obesity in mice causes cognitive decline associated with inhibition of neurogenesis in hippocampus.

## 1 Introduction

Not only diet but also stress can trigger obesity (Tomiyama, 2019; Zhou et al., 2022), and stress can also increase risk of depression (Jiang et al., 2022), anxiety (De Gregorio et al., 2022) and cognitive impairment (Ma et al., 2023), which obesity can increase further (Fulton et al., 2021). Most of the literature on stress-induced obesity in animal models was obtained using animals on high-fat diets (Jene et al., 2021; Lama et al., 2022; Noronha et al., 2019; Zhu et al., 2020). As a result, many insights about the effects of stress on cognitive function, mood or mental health may be confounded by the influence of diet. In our laboratory, we observed that chronic mild stress can induce obesity in 10–20% mice even when they are maintained on a normal diet (Supplementary Figure S1), suggesting that we could explore the influence of stress on obesity and cognitive function without the complicating influences of diet.

Therefore, the present study compared subsets of mice whose body weight increased or decreased significantly in response to chronic mild stress, even though they were maintained on a normal diet. We wanted to explore whether chronic stress capable of inducing obesity could cause several pathologies observed in the absence of obesity, particularly chronic inflammation, psychotic and mood disorders and cognitive impairment (Baumeister et al., 2016; Holsboer and Ising, 2008; Lasikiewicz et al., 2013), and further explored the underlying mechanisms of stress-induced obesity by focusing on the hypothalamic–pituitary–adrenal (HPA) axis and gut microbiome. In response to stress, the HPA axis is activated in order to trigger adaptive behaviors and re-establish homeostasis (Dalile et al., 2022; Micale and Drago, 2018). Over-activation of the HPA axis causes dysregulation of hormone secretion, which contributes to stress-induced obesity (Schinke et al., 2017; Schinke et al., 2022; Vicennati et al., 2014). Dysregulation in the gut microbiome can lead to obesity by altering the absorption of nutrients in the host, modifying metabolic pathways in the host and secreting bacterial metabolites that target the brain directly via vagal stimulation or indirectly through immune-neuroendocrine mechanisms involving corticotropin-releasing hormone, adrenocorticotropin and corticosterone (Geng et al., 2022; Tseng and Wu, 2019; Wang et al., 2020).

Stress-induced dysregulation of the gut microbiome can permeabilize the gut, activate microglia and induce neuroinflammation (Braniste et al., 2014; Geng et al., 2019; Spadoni et al., 2015), ultimately impairing neurogenesis in the hippocampus and cognitive function (Ma et al., 2023; He et al., 2024; He et al., 2024). Alterations in the gut microbiome can activate the HPA axis (Misiak et al., 2020) and, conversely, activation of the HPA axis can impact the gut microbiota (Rusch et al., 2023; O'Mahony et al., 2020). These observations were made in animals that were not obese or that were made obese through a high-fat diet. Here we examined whether the same was true for animals with stress-induced obesity despite being on a normal diet.

## 2 Materials and methods

### 2.1 Animals

Male C57BL/6 mice, 7 weeks old and free of specific pathogens, were purchased from Chengdu Dashuo Laboratory Animals (Chengdu, China), housed individually and assigned unique numbers. The mice were acclimated for a week before the experiment. The mice were housed individually under standard condition (23°C ± 2°C, 50% ~ 70% humidity, on a 12–12 h light–dark cycle with lights on at 07,00 and lights off at 19,00, with access to food and water *ad libitum*) and were allowed to habituate to laboratory conditions for 1 week prior to the experiments. Weight-matched animals were randomly divided to control group (Ctrl, *n* = 12 mice) and chronic mild stress group (CMS, *n* = 30 mice). Mice from CMS group were exposed to chronic mild stress as described in 2.3 section. Control mice were not subjected to any stress and housed in their cages similar to the habituation period.

All animal experiments were approved by the Institutional Animal Care and Use Committee at the Guizhou University of Traditional Chinese Medicine (20,231,215,001, Guiyang, China). All experiments were performed in accordance with ARRIVE guidelines 2.0 (2020) (Percie et al., 2020) and with the “Guidelines for the Care and Use of Laboratory Animals” from the United States National Institutes of Health.

### 2.2 Chronic mild stress

Mice from CMS group were exposed to chronic mild stress for 4 weeks as described (Agudelo et al., 2014). Every day, mice in the stressed group were exposed to 2–3 of the following stressors in random order: tilting of the cage (45°, 24 h), clamping of the tail (10 min), shaking of the cage (30 min), continuous light (24 h), continuous darkness (24 h), restraint (2 h), strobe light (12 h), empty bottle (6 h), wet bedding (12 h), empty cage (24 h), and scent stimuli (mice were placed in a pepper-smelled chamber for 15 min).

During the 4 weeks, all animals were weighed every Wednesday and Sunday at 15:00. Once weekly, they were deprived of food and water for 12 h and assessed in the sucrose preference test (see section 2.3.1 below). Also once weekly, their physical state was assessed in terms of the coat score assay as described (Cao et al., 2013). The fur in the following seven body areas was assigned 0 point if it was unkempt (un-groomed) or 1 point if it was well-groomed: head, neck, forepaws, dorsal coat, ventral coat, hind paws and tail. The seven scores were summed to obtain the total coat score.

After 4 weeks, animals whose body weight was at least one standard deviation smaller or larger than that of unstressed control animals were classified as showing stress-induced weight loss (WL) or gain (WG), respectively. These two groups were confirmed by body mass index and serum lipid level, and compared in several ways such as based on the fecal microbiome, systemic behavior, hormone levels, peripheral and neural cytokines and hippocampal neurogenesis (see below).

### 2.3 Behavioral testing

#### 2.3.1 Sucrose preference test

This test was performed as described (Zhang et al., 2021). Animals were deprived of water or food for 12 h, then given *ad libitum* access for 12 h to two identical bottles, one containing water and the other 1% sucrose solution. The bottle positions were switched daily to avoid side bias. Both bottles were weighed before and after the 48-h period. Sucrose preference was calculated as follow: sucrose preference (%) = sucrose consumed (g) / [sucrose consumed (g) + water consumed (g)] × 100%.

#### 2.3.2 Open field test

After 4 weeks of chronic mild stress, this test was performed in a box measuring 40 cm × 40 cm × 30 cm as described (Liu et al., 2022). Each mouse was placed in a corner at the start of the test and recorded for 5 min by a camera located above the box. Before each mouse was tested, the feces and urine left by the previous mouse were clean and deodorized with 75% ethanol. Open field lighting is produced by energy-saving lamps with a light intensity of about 200 Lux, and the noise is controlled below 65 db. The OFT100 system for tracking and analysis (Taimeng Tech, Chengdu, China) determined the number of entries into the central zone (covering 50% of the total area), the time spent in the central zone and the total distance traveled.

#### 2.3.3 Tail suspension test

This test was performed as described (Zhang et al., 2021). Mice were elevated 35 cm above the ground by securing their tails with adhesive tape. Mice were recorded for 6 min, and the time spent immobile during the last 5 min was measured using YHTSTM software (LabState ver 2.0, Yihong Tech, Wuhan, China).

#### 2.3.4 Forced swimming test

This test was performed as described (Zhang et al., 2021). Mice were placed for 10 min into a cylinder 25 cm high with a diameter of 10 cm, which was filled up with water at 24°C to two-thirds of the full volume. Then the animals were returned to their cages. After 24 h, the animals were placed again in the cylinder for 6 min, and the duration of immobility during the last 5 min was determined using YHTSTM software (LabState ver 2.0, Yihong Tech).

#### 2.3.5 Tests of novel location recognition and novel object recognition

These tests measure the extent to which animals have retained their innate preferences for new objects and locations and are therefore widely used to assess hippocampus-dependent learning and memory (Ennaceur and Delacour, 1988). The experimental environment was the same as in the open field experiment.

In the training stage of the test of novel location recognition, two identical objects (non-porous/cleaned to make sure there’s no scent marking) were placed in different positions in the box, then mice were placed in the central area of the box and allowed to freely explore the two objects for 10 min and thereby develop a memory of the location of the two objects. Before each mouse was tested, the feces and urine left by the previous mouse were clean and deodorized with 75% ethanol. The animals were returned to their cages, 6 min later, the position of one of the objects was randomly changed, and the mice were returned to the box and allowed to explore freely for another 10 min. The number of times that mice sniffed the object in the new location and the total time spent sniffing it (as a proportion of the total exploration time) were recorded. Greater proportion of exploration time that was spent sniffing objects in the new location was taken to indicate stronger location memory.

After animals completed the test of novel location recognition, two objects identical in shape, color, and odor were introduced into the box. Then mice were placed in the central area of the box and allowed to freely explore the two objects for 10 min. The animals were returned to their cages, 30 min later, then one of the original objects and a novel object (differing in the original objects) were placed in the box. The mice were returned to the box and allowed to explore freely for another 10 min. The time spent exploring each object was determined during each session. Greater proportion of exploration time that was spent sniffing the new objects was taken to indicate stronger object memory.

### 2.4 Analysis of fecal microbiomes

All fecal samples were collected at the same time (19:00) to avoid circadian influences on the microbiome. Fecal samples were examined instead of cecum contents to assess whether differences WL, WG or control. Fecal samples from 7 control animals, 7 WL animals and 6 WG animals were subjected to microbiome analysis. The composition of the fecal microbiome was analyzed using 16S rRNA sequencing, which was performed by Majorbio Bio-Pharm Technology (Shanghai, China). Sequencing experiments involved the following steps: DNA extraction, PCR amplification and product purification, MiSeq library construction, and MiSeq sequencing. PCR amplification was carried out using TransStart FastPfu DNA Polymerase (TransGen Biotech, Beijing, China) and an ABI GeneAmp® 9,700 system (Thermo Fisher Scientific, Wilmington, DE, United States). Bacterial DNA fragments were amplified using forward primer-338 (5′-ACTCCTACGGGAGGAGCAG-3′) and reverse primer-806 (5′-GCACTACHVGGGTWTCTAAT-3′). The DNA was quantified using a Nanodrop spectrophotometer (Thermo Fisher Scientific), and paired end sequencing of the V3-V4 region of 16S rRNA was performed as previously described (Pearson-Leary et al., 2020) using an Illumina MiSeq PE300 platform (Illumina, San Diego, United States). The raw sequencing reads were deposited into the NCBI Sequence Read Archive (BioProject ID PRJNA1047189).

The 300 bp reads were truncated at any site receiving an average quality score of <20 over a 50 bp sliding window, and the truncated reads shorter than 50 bp were discarded, reads containing ambiguous characters were also discarded. Only overlapping sequences longer than 10 bp were assembled according to their overlapped sequence. Barcode accurately matched and separated samples, primers adjusted the sequence direction, and selected sequences with mass greater than 20 (that is, the accuracy of 99%) for downstream analysis. In order to remove the primer cleanly, set the primer mismatch rate to 0.15. Unexcised sequences of primers are discarded directly to prevent false operational taxonomic units (OUTs). Then the optimized sequences were clustered into OTUs using UPARSE 7.1 with 97% sequence similarity level. The most abundant sequence for each OTU was selected as a representative sequence.

Bioinformatic analysis of the gut microbiota was carried out using the Majorbio Cloud platform.^1^ Based on the OTUs information, rarefaction curves and alpha diversity indices including observed OTUs, Ace, Chao richness, Simpson and Shannon index were calculated with Mothur v1.30.1. The similarity among the microbial communities in different samples was determined by principal coordinate analysis (PCoA) based on Bray–curtis dissimilarity using Veganv2.5–3 package. The PERMANOVA test was used to assess the percent-age of variation explained by the treatment along with its statistical significance using Vegan v2.5–3 package.

### 2.5 Analysis of serum lipids, hormones and cytokines

Five mice were randomly selected from each group. These mice were anesthetized (isoflurane 4% induced anesthesia and 2% maintained anesthesia in mice), cardiac puncture was performed to collect the whole blood, and the serum was collected by centrifuging at 4°C 1500 r / min for 15 min. The serum was assayed for low-density lipoprotein cholesterol (LDL), high-density lipoprotein cholesterol (HDL), total cholesterol (TC), triglyceride (TG), corticotropin-releasing hormone (CRH), adrenocorticotropin (ACTH) and corticosterone (CORT), interleukin-6 (IL-6), tumor necrosis factor *α* (TNF-α) and interleukin-10 (IL-10) (Beyotime. Shanghai, China) in strict accordance with the manufacturer’s instructions. These kits obtained from the Guizhou Lvmeng Weiye Biotechnology Co., LTD (Guizhou, China).

After blood collection, all mice were perfused with 0.9% NaCl. Brains were removed and divided into two hemispheres. The intact hippocampus was removed from left hemisphere and used for transcriptome sequencing and cytokine detection while the right hemisphere was used for immunohistochemical staining.

### 2.6 Analysis of cytokines in the hippocampus

Hippocampi were dissociated and lysed in RIPA lysis buffer (catalog no. R0010, Solarbio, Beijing, United States) containing phenylmethanesulfonyl fluoride (catalog no. IP0280, Solarbio). Lysates were centrifuged for 15 min at 1000 *g* at 4°C, the concentration of total protein in the supernatant was determined using a commercial bicinchoninic acid kit (Boster, Wuhan, China), then samples were diluted to a final total protein concentration of 1 g/mL. The pro-inflammatory factor IL-1β was quantitatively analyzed by the double antibody sandwich method (Piao et al., 2023). The diluted samples were added to 96-well plates coated with anti-mouse IL-1β antibody, labeled with biotin, and incubated with avidin peroxidase complex labeled antibody. OD values were detected at 450 nm and protein expression was calculated. The manufacturer-specified detection limit was 2 pg. / mL.

### 2.7 Hippocampal gene expression

The hippocampal transcriptome was analyzed as described (Zhang et al., 2021). Total RNA of hippocampus was extracted using TRIzol® (Invitrogen, Carlsbad, CA, United States) according to the manufacturer’s instructions. RNA was sequenced by Majorbio Bio-Pharm Technology, and all sequences were uploaded to the NCBI Sequence Read Archive under BioProject ID PRJNA1046681.

### 2.8 Tissue preparation and immunohistochemistry

The right hemispheres from each group mice were fixed with 4% paraformaldehyde for 48 h, dehydrated, frozen, sliced, thoroughly washed with 0.5% Triton X-100 for 15 min, blocked with 10% donkey serum for 1 h, and incubated at 4°C overnight with monoclonal antibody against Iba1 (1:400; Abcam, Cambridge, United Kingdom) to label microglia or against glial fibrillary acidic protein (1,400; Cell Signaling Technology, Danfoss, MA, United StAtes) to label astrocytes. Sections were washed three times with phosphate-buffered saline (PBS), incubated with secondary antibody in the dark for 2 h, and counterstained with DAPI (1,10,000; Roche, Switzerland).

To measure hippocampal neurogenesis, all mice received intraperitoneal injections of 5-bromo-2-deoxyuridine (5 mg/mL BrdU in 0.9% saline; Sigma, St. Louis, MO, USA) at a dose of 50 mg/kg once every 12 h for 7 days. At 48 h after the last injection, mice were perfused and brains were taken, and one of every six sequential tissue slices containing hippocampus was permeabilized with 0.5% Triton X-100 in PBS for 15 min, incubated in 2 N HCl for 28 min at 37°C, washed twice with 0.1 M borate buffer (pH 8.5) for 10 min each, blocked in 10% donkey serum for 2 h, and incubated with primary antibody against doublecortin (1:300, Cell Signaling Technology) to label immature neurons, BrdU (1:400, Cell Signaling Technology) to label proliferating neural stem/progenitor cells, or nuclear protein NeuN (1:400, Cell Signaling Technology) to label neurons. Then sections were incubated for 2 h at room temperature with Alexa-conjugated secondary antibody (1:300; Invitrogen, Carlsbad, CA, United States), followed by counterstaining with DAPI for 5 min.

In both types of staining, sections were imaged under a fluorescence microscope (Olympus IX 73, Tokyo, Japan), and images were analyzed using Image J 1.45 (United States National Institutes of Health, Bethesda, MD, United States). Percentages of total area that were positively stained or numbers of cells were averaged across five micrographs at 40× magnification per mouse.

### 2.9 Statistical analysis

Data were analyzed for normal distribution using the Shapiro–Wilk test, and reported as mean ± standard error. Differences among unstressed control mice and those showing stress-induced weight loss or gain were assessed for significance using one-way analysis of variance, followed by Tukey’s *post hoc* test for multiple comparisons. All statistical analyses were performed using GraphPad Prism 8.0 (GraphPad, San Diego, CA, United States). Results associated with *p* < 0.05 were considered significant.

## 3 Results

### 3.1 Stress-induced obesity in mice involves cognitive impairment

**Figure 1:**
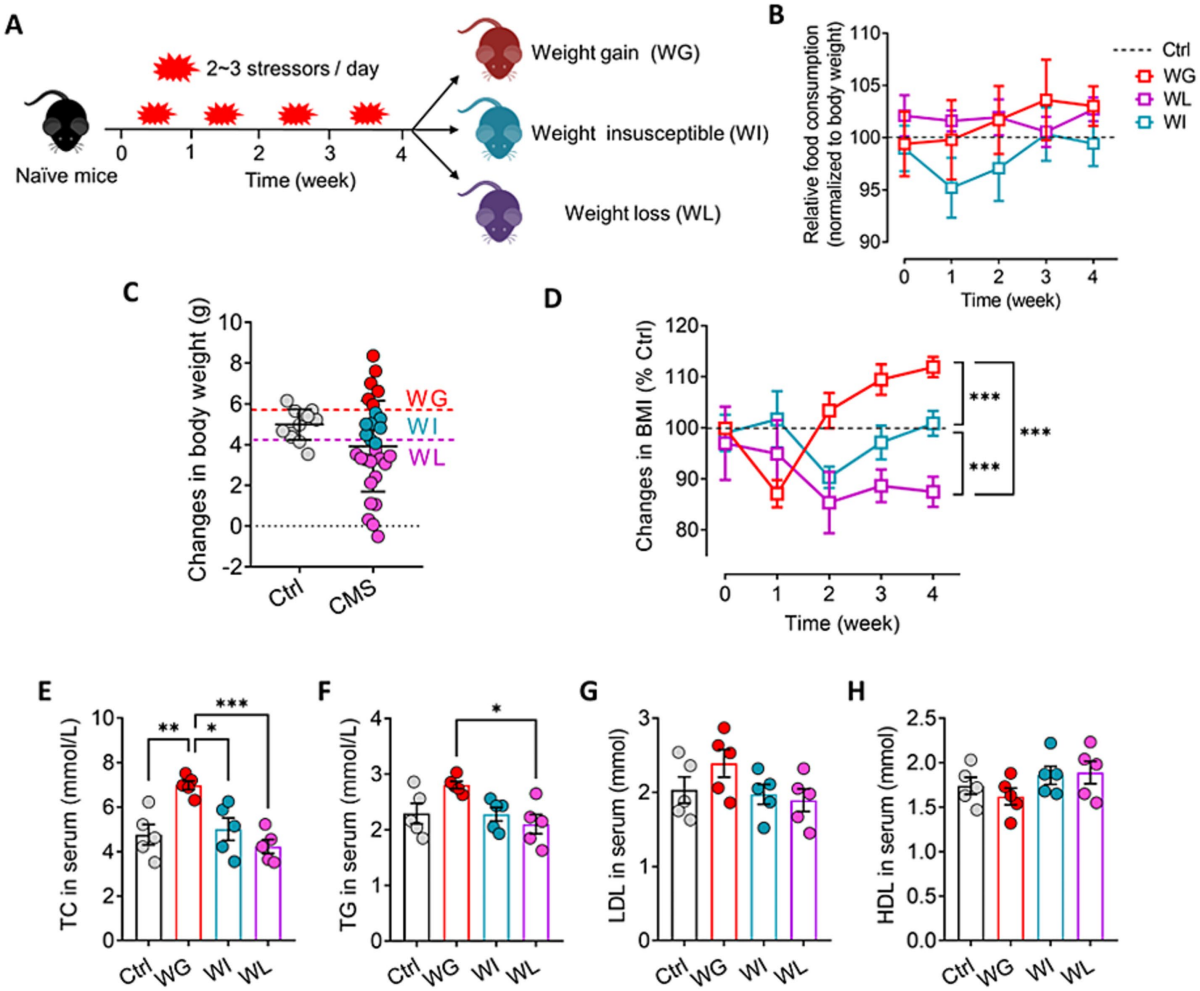
Screening of mice that exhibited stress obesity. **(A)** Schematic for identification of mice showing weight gain (WG), weight insusceptible (WI) or weight loss (WL) after stress exposure. **(B)** Changes in food consumption of WG, WI or WL mice during 4 weeks of CMS exposure. Levels were normalized to their body weight. **(C)** Mice were exposed to chronic mild stress (CMS) for 4 weeks, and animals were classified as WG, WI or WL on the basis of their body weight change. Ctrl, control animals never subjected to CMS. **(D)** Changes in body mass index (BMI) of WG, WI or WL mice during 4 weeks of CMS exposure. Levels were normalized to control mice. **(E–H)** Levels of low-density lipoprotein (LDL), high-density lipoprotein (HDL), total cholesterol (TC), and triglyceride (TG) in serum. Results are shown for triplicate samples from five animals per condition. Data are mean ± standard error of the mean (SEM). Quantitative results come from 6 to 15 animals per condition for panel C and D, and from 5 animals per condition for panel E–H. *p < 0.05; **p < 0.01; ***p < 0.001, based on one-way ANOVA followed by Tukey’s multiple-comparisons test.

Mice were exposed to CMS for 4 weeks and divided into weight gain (WG), weight loss (WL) and weight insusceptible sub-group based on their body weight changes relative control mice (Figures 1A,B). We traced the changes in body mass index and food consumption of each sub-group of animals during the CMS period, and found that WG mice showed increase and WL mice showed decrease in their body weight from week-2 to week-4 when compared with control mice (Figure 1C). However, there was no significant difference in food consumption among control, WG, WI, and WL mice during 4 weeks of CMS exposure (Figure 1D).

Chronic mild stress led to significant weight gain or loss in subsets of animals starting halfway through the four-week stress paradigm, even though all animals consumed similar amounts of normal diet throughout the paradigm (Figures 1A–D). Animals showing stress-induced weight gain also showed up-regulation of total cholesterol and triglyceride in serum, consistent with obesity (Du et al., 2019; Figures 1E–H). There was no significant difference in the above indexes between weight insusceptible sub-group and the control group.

**Figure 2:**
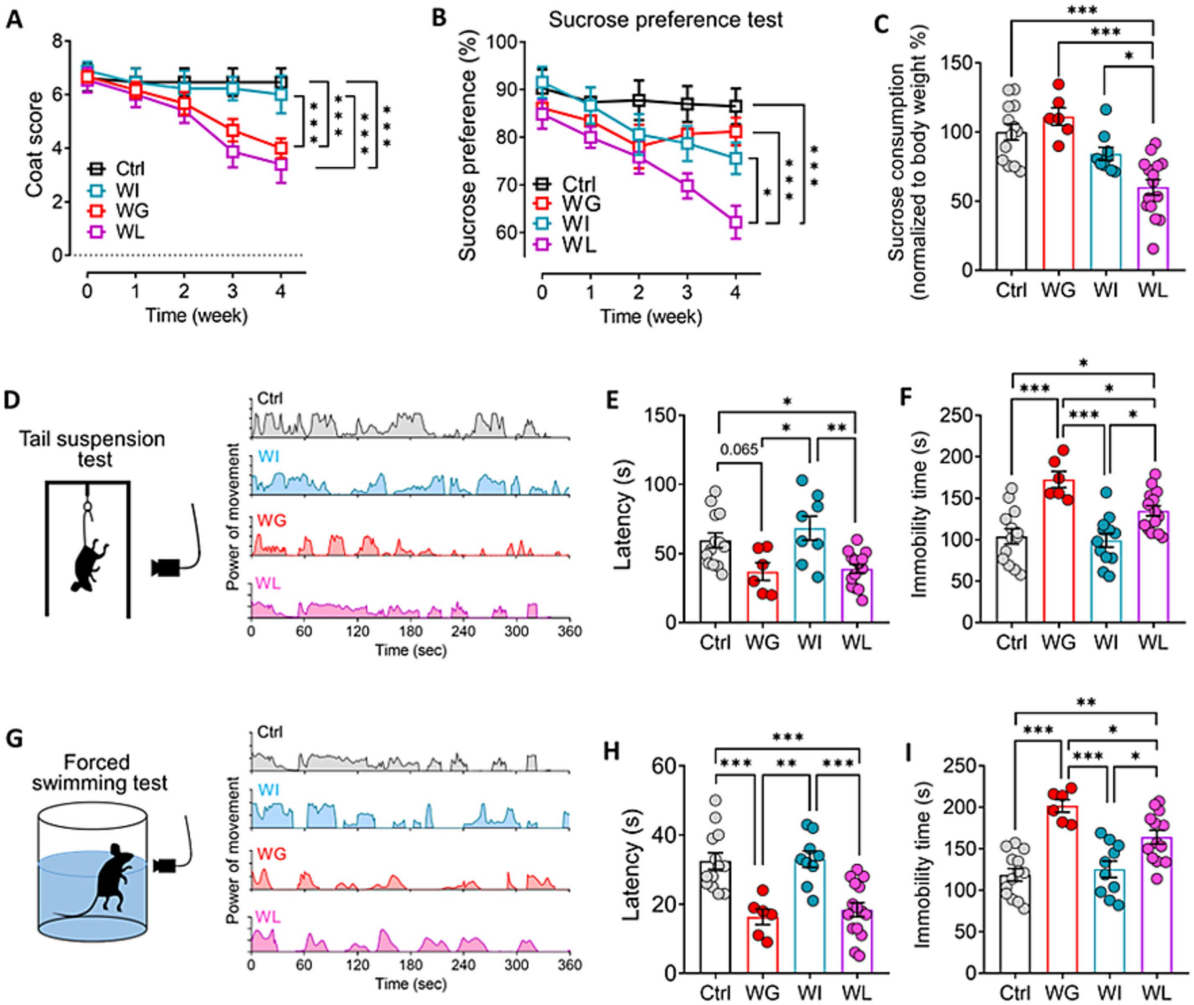
Differences between mice showing weight gain, insusceptible or loss after stress exposure in depressive-like behaviors. **(A,B)** Coat score and sucrose preferent of control (Ctrl), weight gain (WG), weight insusceptible (WI) or weight loss (WL) mice. Ctrl, control animals never subjected to CMS. **(C)** Sucrose consumption of mice in sucrose preference test. Levels were normalized to their body weight. **(D)** Power of movement from Ctrl, WG, WI or WL mice in tail suspension test during 6 min. **(E,F)** Quantization of **(E)** latency and **(F)** immobility time of Ctrl, WG, WI or WL mice in tail suspension test. **(G)** Power of movement from Ctrl, WG, WI or WL mice in forced swimming test during 6 min. **(H,I)** Quantization of **(E)** latency and **(F)** immobility time of Ctrl, WG, WI or WL mice in forced swimming test. Data are mean ± standard error of the mean (SEM). Quantitative results come from 6–15 animals per condition. *p < 0.05; **p < 0.01; ***p < 0.001, based on one-way ANOVA followed by Tukey’s multiple-comparisons test.

Chronic mild stress induced depression- and anxiety-like symptoms in mice, regardless of whether they showed significant weight gain or loss (Figure 2; Supplementary Figure S2). However, the detailed nature of the symptoms differed between the two groups: animals that gained weight performed worse in the forced swimming and tail suspension tests than animals that lost weight, indicating greater behavioral despair (Porsolt et al., 2001); conversely, animals that lost weight showed lower sucrose preference, indicating greater anhedonia (Liu et al., 2018).

**Figure 3:**
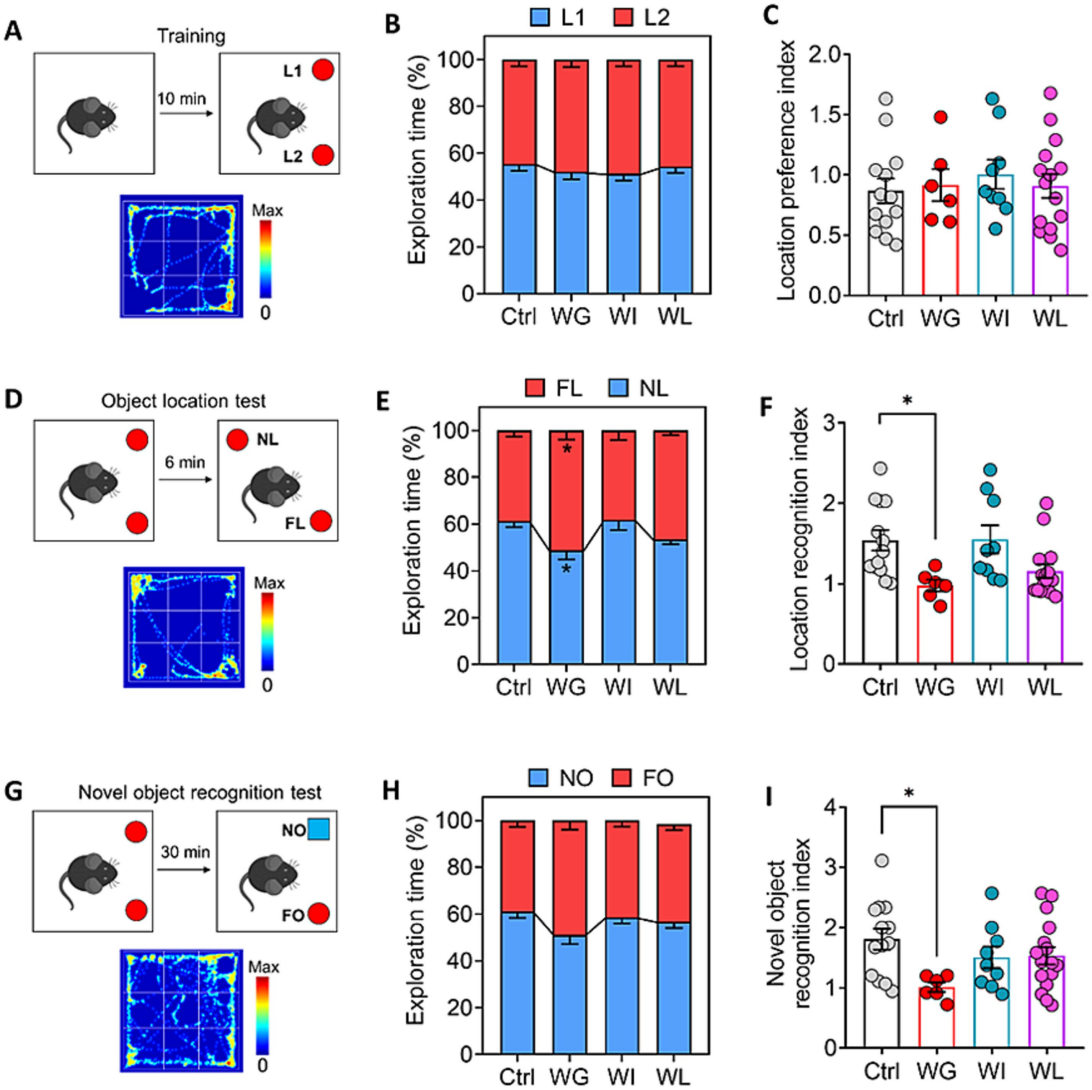
Differences between mice showing weight gain, insusceptible or loss after stress exposure in cognitive function. **(A)** Schematic for location preference test. Heatmap shows the exploration track of control mice on the object in location 1 (L1) and location (L2). **(B)** Quantization of time spent exploring objects in L1 and L2 of control (Ctrl), weight gain (WG), weight insusceptible (WI) or weight loss (WL) mice. Ctrl, control animals never subjected to CMS. **(C)** Location preference index was compared between the groups.**(D)** Schematic for object location test. Heatmap shows the exploration track of control mice on the object in novel location (NL) and familiar location (FL). **(E)** Quantization of time spent exploring objects in NL and FL of Ctrl, WG, WI or WL mice. **(F)** Location recognition index was compared between the groups. **(G)** Schematic for object location test. Heatmap shows the exploration track of control mice on the novel object (NO) and familiar object (FO). **(H)** Quantization of time spent exploring objects in NO and FO of Ctrl, WG, WI or WL mice. **(I)** Novel object recognition index was compared between the groups. Data are mean ± standard error of the mean (SEM). Quantitative results come from 6–15 animals per condition. *p < 0.05; **p < 0.01; ***p < 0.001, based on one-way ANOVA followed by Tukey’s multiple-comparisons test.

Chronic mild stress did not alter the animals’ natural lack of location preference (Figures 3A–C), but it did significantly reduce the time that animals with weight gain spent exploring objects in novel locations (Figures 3D–F) and exploring novel locations (Figures 3G–I). These results link stress-induced obesity to impaired memory of objects and locations. These behavioral changes were not observed in the weight insusceptible sub-group.

Taken together, these behavioral experiments suggest that chronic mild stress can induce depression- and anxiety-like behaviors in animals regardless of whether it also leads to significant weight gain, whereas such stress impairs cognitive function only when it leads to significant weight gain.

### 3.2 Stress-induced obesity in mice involves hyperactivation of the HPA axis and alterations in the gut microbiome

**Figure 4:**
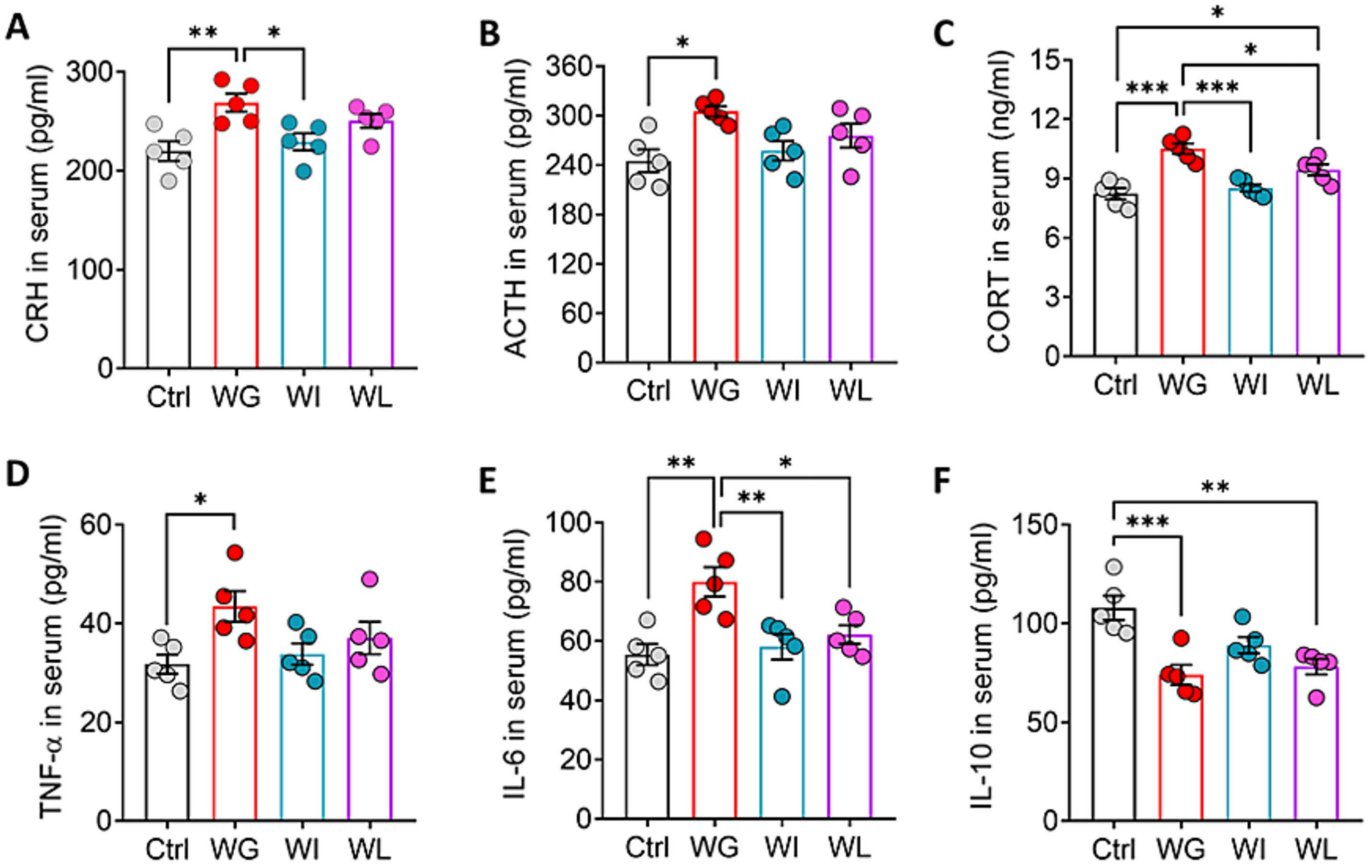
Mice that exhibited stress obesity were accompanied by hyperactivation in HPA axis and inflammatory responses. **(A–C)** Levels of corticotropin-releasing hormone (CRH), adrenocorticotropic hormone (ACTH) and corticosterone (CORT) in serum. Results are shown for triplicate samples from five animals per condition. **(D–F)** Levels of tumor necrosis factor-*α* (TNF-α), interleukin-6 (IL-6) and interleukin-10 (IL-10) in serum. Results are shown for triplicate samples from five animals per condition. Data are mean ± standard error of the mean (SEM). Quantitative results come from 5 animals per condition. *p < 0.05; **p < 0.01; ***p < 0.001, based on one-way ANOVA followed by Tukey’s multiple-comparisons test.

Animals showing stress-induced weight gain also showed upregulation of corticotropin-releasing hormone, adrenocorticotropin and corticosterone in serum, consistent with HPA activation (Stout et al., 2002; Figures 4A–C). Animals showing stress-induced weight loss showed upregulation of corticosterone relative to unstressed controls, but the levels were not as high as in mice with stress-induced weight gain. Consistent with the link between HPA activation and upregulation of pro-inflammatory cytokines (Ensminger et al., 2021), we found that animals showing stress-induced weight gain also showed upregulation of tumor necrosis factor-*α* and interleukin-6 in serum (Figures 4D–F). This upregulation was not observed in animals showing stress-induced weight loss and weight insusceptible. Both WG and WI animals showed downregulation of the anti-inflammatory cytokine interleukin-10. There was no significant difference in the above indexes between weight insusceptible sub-group and the control group.

Since the WI group showed no significant difference from the control group in behavioral test results such as anxiety, depression and cognitive function, and the expression levels of obesity-related indicators and pro-inflammatory factors. Therefore, in the subsequent analysis, we focused on WG and WL groups.

**Figure 5:**
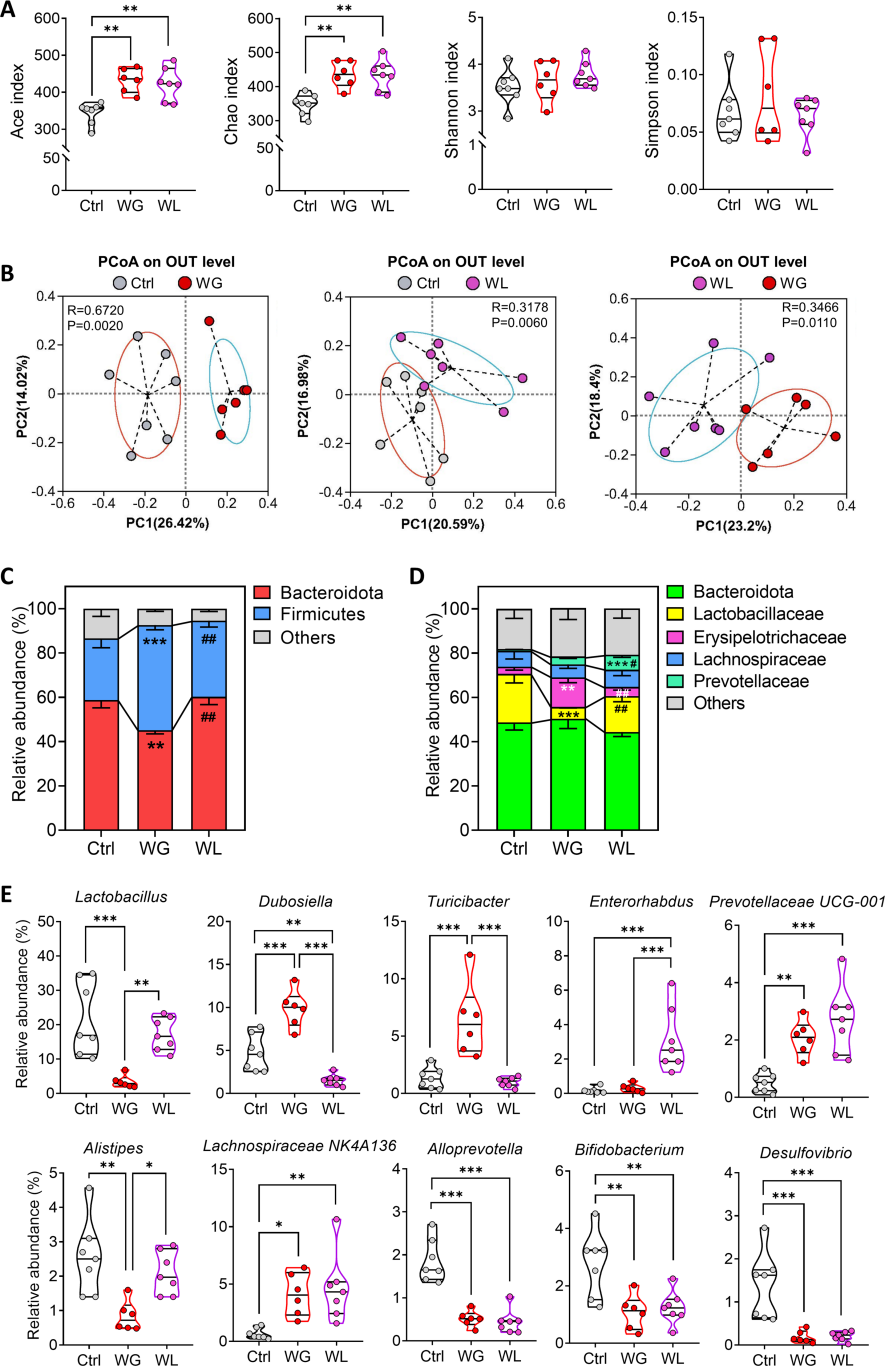
Differences in gut microbiome composition between mice showing weight gain or loss after stress exposure. **(A,B)** Alpha diversity and beta diversity of fecal microbiota from control (Ctrl), weight gain (WG), weight insusceptible (WI) or weight loss (WL) mice. Ctrl, control animals never subjected to CMS. OTU, operational taxonomic unit; PCoA, principal component analysis; PC, principal component. **(C,D)** Relative abundances of **(C)** phyla and **(D)** families in fecal microbiota. **(E)** Differences in relative abundance of individual genera. Quantitative results come from 6 to 7 samples per condition. Data in panels **A** and **E** are shown as violin plots. The horizontal line within the violin plots represents the median, upper, and lower quartiles. The width of the plot depicts the density and distribution shape of the data points. *p < 0.05, **p < 0.01, ***p < 0.001, based on one-way ANOVA with Tukey’s multiple-comparisons test. Data in panel **C** and **D** are mean ± standard error of the mean (SEM), **p < 0.01, ***p < 0.001 vs. Ctrl group, ^#^*p* < 0.05, ^##^*p* < 0.01 vs. WG group, based on one-way ANOVA with Tukey’s multiple-comparisons test.

Fecal bacteria from animals showing stress-induced weight gain or loss showed significantly higher Ace and Chao indices than those from unstressed controls (Figure 5A), suggesting that stress increased the richness of gut microbiota. In contrast, stress did not significantly affect Shannon or Simpson indices of fecal bacteria, suggesting minimal effects on the alpha diversity of gut microbiota. Principal component analysis of variation in non-phylogenetic Bray–Curtis metrics showed that the structure of the bacterial community differed significantly between animals showing stress-induced weight loss or gain, and between each of those groups and unstressed controls (Figure 5B).

At the phylum level, animals with stress-induced weight gain showed a significant increase in abundance of Firmicutes bacteria but significant decrease in Bacteroidota bacteria, while the opposite was observed in animals with stress-induced weight loss (Figure 5C). At the family level, animals with stress-induced weight gain showed a significant increase in abundance of *Erysipelotrichaceae, Lactobacillaceae* and *Prevotellaceae,* but significant decrease in *Lactobacillaceae* bacteria; the opposite was observed in animals with stress-induced weight loss (Figure 5D).

At the genus level, animals with stress-induced weight gain showed a significant increase in *Dubosiella*, while the opposite was observed in animals with stress-induced weight loss (Figure 5E). Those with weight gain showed a significant increase in abundance of *Turicibacter* bacteria but a significant decrease in abundance of *Lactobacillus* and *Alistipes* bacteria. Animals with weight loss showed a significant increase in abundance of *Enterorhabdus* bacteria. In both groups of mice, stress induced similar changes in abundances of *Alloprevotella*, *bifidobacterium, Desulfovibrio, Prevotellaceae UG-001 and Lachnospiraceae NK4A136 bacteria.*

### 3.3 Stress-induced obesity in mice involves glial hyperactivation and inhibition of neurogenesis in hippocampus

**Figure 6:**
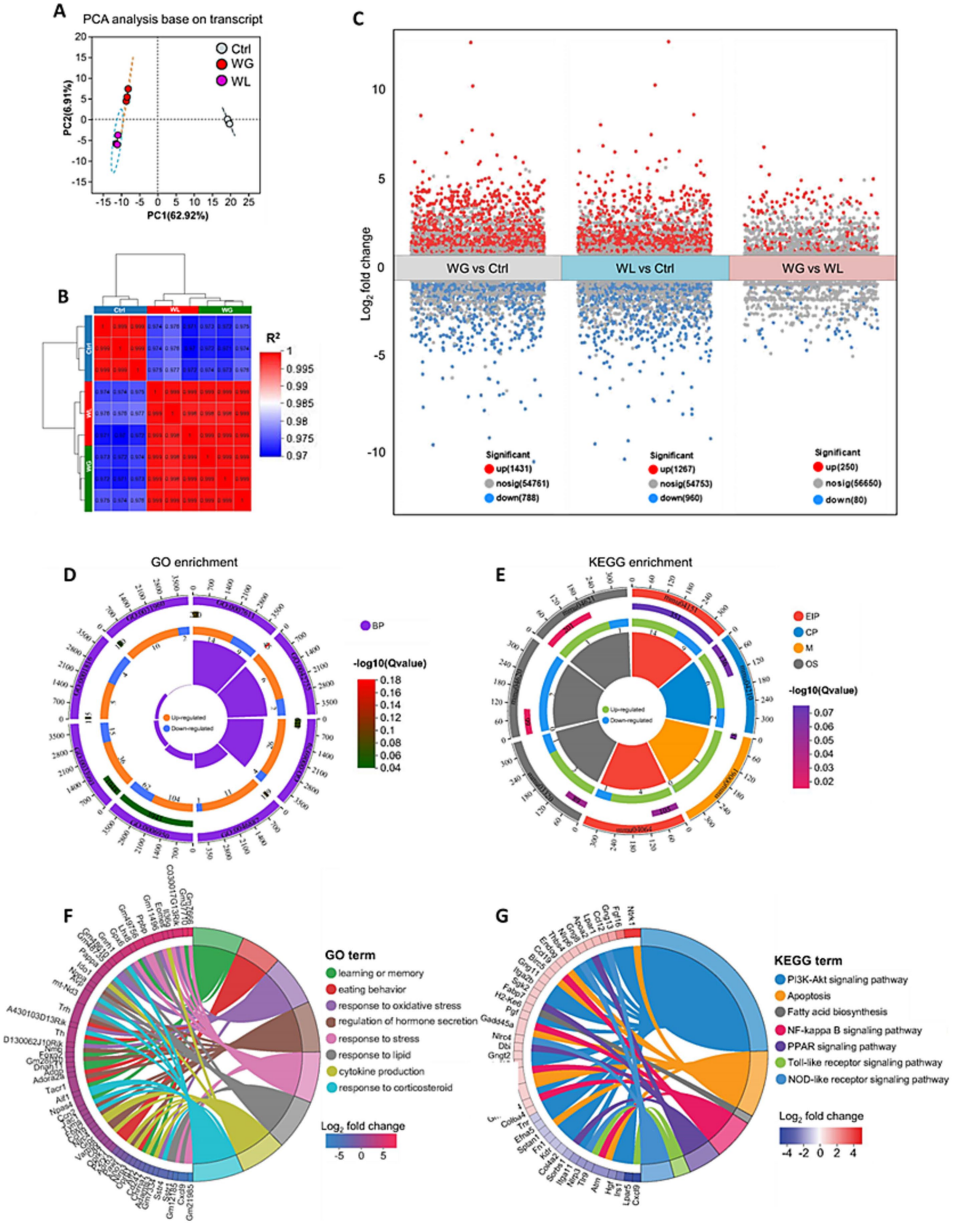
Differences between mice showing weight gain, insusceptible or loss after stress exposure in gene expression in the hippocampus. **(A)** Principal component analysis to identify clusters of transcriptomes of hippocampus from control (Ctrl), weight gain (WG) or weight loss (WL) mice. PC, principal component. **(B)** Correlation analysis between samples. **(C)** Volcano plot of differentially expressed genes. Significantly upregulated genes are shown as red dots; significantly downregulated genes, as blue dots. **(D,E)** The loop map of gene enrichment shows GO enrichment (left) and Kyoto Encyclopedia of Genes and Genomes (KEGG) enrichment (right) of up-regulated and down-regulated genes between WG and WL mice. **(F,G)** Circos plot showing the connectivity map derived from the pairwise comparison of transcriptome datasets. The connectivity between DEGs (left) and GO term or KEGG pathway (right) are shown in different color. Each line represents a pairwise dataset overlap, which was determined using Gene Set Enrichment Analysis and filtered by *p* < 0.05 and normalized enrichment score > 1.5. The change in the expression of DEGs was quantified as the log2 fold change.

**Figure 7:**
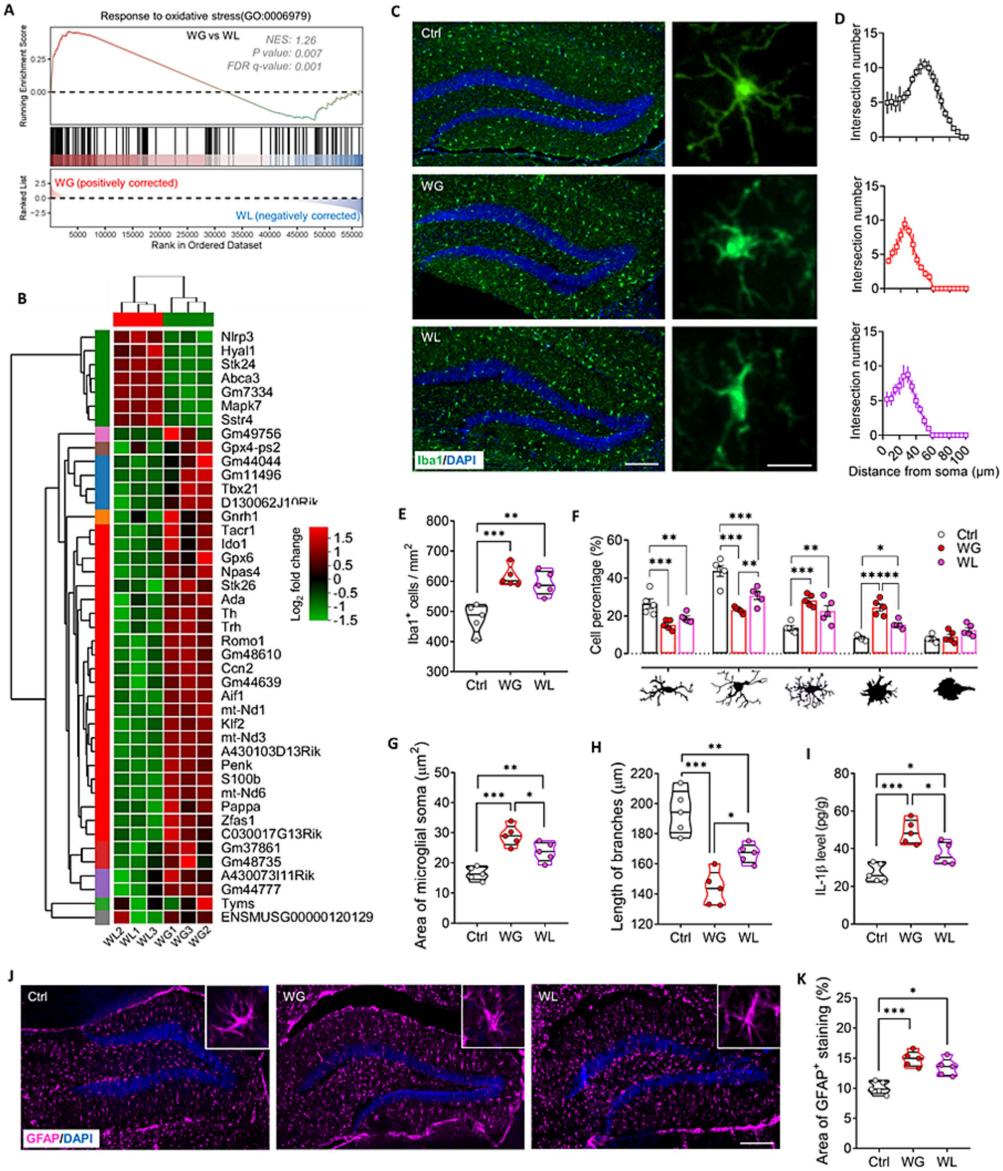
Mice that exhibited stress obesity were accompanied by hyperactivation in hippocampal microglia. **(A)** Gene set enrichment analysis, indicating enrichment of processes related to response to oxidative stress (GO:0006979) in the hippocampal transcriptome of weight gain (WG) mice compared to that of weight loss (WL) animals after stress exposure. **(B)** Hierarchical cluster analysis of enriched differentially expressed genes related to response to oxidative stress. **(C)** Representative sections of hippocampal dentate gyrus from mice that were classified as showing WG or WL or from control animals never subjected to stress (Ctrl) were immunostained against **“**ionized calcium binding adapter molecule 1” (Iba1) as a marker of microglia. Scale bar, 100 μm. The enlarged graphs on the right are the typical morphology of microglia in hippocampus of Ctrl, WG or WL mice. Scale bar, 15 μm. **(D)** The number of intersections of microglial branches were evaluated by Sholl analysis in the hippocampus of Ctrl, WG or WL mice. **(E)** Quantification of the number of microglia hippocampus of Ctrl, WG or WL mice. Results come from five slices of hippocampal dentate gyrus (at 40 × magnification) from each of five mice per condition. Each dot represents the average of all micrographs for one mouse. **(F)** Quantification of the proportions of microglia in the hippocampus that showed, from *left* to *right,* longitudinal branching, radial branching, hyper-branching, or compact and ameboid form. **(G,H)** Quantification of the area of microglial soma and length of microglial branches in hippocampus. **(I)** Assay of levels of interleukin (IL)-1βin hippocampus. Quantitative results come from 5 animals per condition. **(J)** Representative sections of hippocampal dentate gyrus immunostained against glial fibrillary acidic protein (GFAP) as a marker of astrocytes. Nuclei were counterstained with DAPI. Scale bar, 100 μm. **(K)** Quantification of GFAP^+^ area in hippocampus of Ctrl, WG or WL mice. Data are mean ± standard error of the mean (SEM). *p < 0.05, **p < 0.01, ***p < 0.001, based on one-way ANOVA with Tukey’s multiple-comparisons test.

Mice with stress-induced weight gain showed upregulation of 1,431 genes and downregulation of 788 genes in the hippocampus relative to mice with stress-induced weight loss (Figures 6A–C). Most of these differentially expressed genes were involved in processes related to learning or memory, eating behavior, stress response, cytokine production, hormone secretion regulation, oxidative stress response and corticosteroid response; and these processes involved apoptosis, fatty acid biosynthesis, and signaling mediated by PI3K-Akt, NF-κB, PPARs, Toll-like receptor and NOD-like receptor (Figures 6D–F). We found that oxidative stress response in the hippocampus was implicated in stress-induced body weight change (Supplementary Figure S3). Gene set enrichment analysis confirmed that genes involved in responding to oxidative stress were upregulated in the hippocampus of animals showing stress-induced weight gain relative to those showing weight loss (Figures 7A,B; Supplementary Figure S2).

Both groups of mice showed significantly larger numbers of hippocampal microglia (detected based on Iba1 expression) than unstressed controls, and a significantly larger proportion of those microglia had enlarged somata, reduced branching and thicker, shorter processes than in controls (Figures 7C–H). These results indicate stress-induced hyperactivation of hippocampal microglia, which was stronger in animals with weight gain than in animals with weight loss. The greater activation in mice with weight gain was associated with higher levels of the pro-inflammatory cytokine interleukin-1β in hippocampus (Figure 7I). Both groups of mice showed significantly larger numbers of hippocampal astrocytes (detected based on expression of glial fibrillary acid protein; Figures 7J,K).

**Figure 8:**
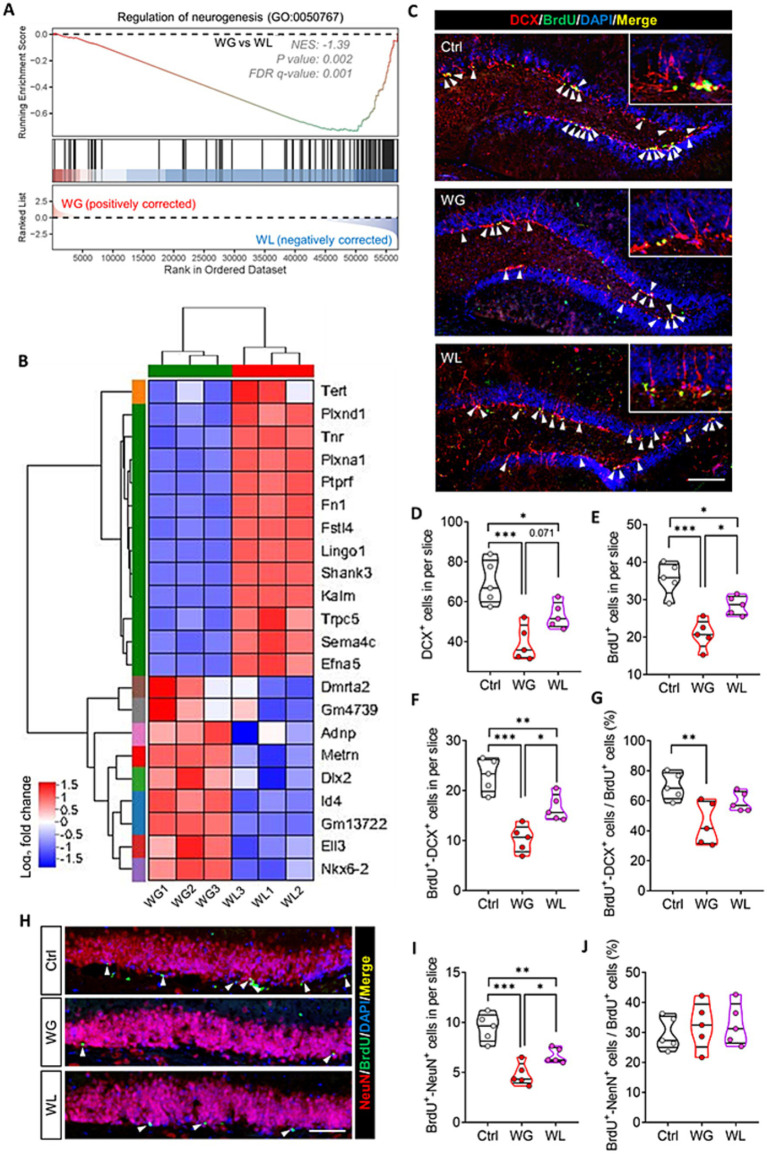
Mice that exhibited stress obesity were accompanied by exaggerated impairment in hippocampal neurogenesis. **(A)** Gene set enrichment analysis, indicating enrichment of processes related to regulation of neurogenesis (GO:0050767) in the hippocampal transcriptome of weight gain (WG) mice compared to that of weight loss (WL) animals after stress exposure. **(B)** Hierarchical cluster analysis of enriched differentially expressed genes related to regulation of neurogenesis. **(C)** Representative sections of hippocampal dentate gyrus from mice that were classified as showing WG or WL or from control animals never subjected to stress (Ctrl) were immunostained against doublecortin (DCX) to label immature neurons cells and BrdU to label proliferating neural stem/progenitor cells. Cells staining for both BrdU and DCX were considered to be newborn neurons. Nuclei were counterstained with DAPI. The *top right corner* shows a higher magnification image. White arrowheads indicate newborn neurons. Scale bar, 100 μm. **(D–G)** Quantification of cell subpopulations in the experiments in panel C. Results come from five slices of hippocampal dentate gyrus (at 40 × magnification) from each of five mice per condition. Each dot represents the average of all micrographs for one mouse. Panel E expresses the extent of neural stem/progenitor cell differentiation into neurons. **(H)** Representative sections of hippocampal dentate gyrus were immunostained against NeuN to label mature neurons or BrdU to label proliferating neural stem/progenitor cells. Cells staining for both BrdU^+^ and NeuN^+^ were considered to be newly mature neurons (white arrowheads). Nuclei were counterstained with DAPI. Scale bar, 50 μm. **(I,J)** Quantification of the experiments in panel **H**. Panel **I** expresses the extent of newborn neuron maturation into mature neurons. Data are mean ± standard error of the mean (SEM). *p < 0.05, **p < 0.01, ***p < 0.001, based on one-way ANOVA with Tukey’s multiple-comparisons test.

Consistent with the link between hyperactivation of hippocampal microglia and impaired neurogenesis in the adult hippocampus (He et al., 2022; Su et al., 2023; Zhang et al., 2020), regulating neurogenesis in the hippocampus was implicated in stress-induced body weight change (Supplementary Figure S4). Genes involved in regulating neurogenesis made up a significantly greater proportion of the genes differentially regulated by stress in animals showing weight gain than in those showing weight loss (Figures 8A,B). Immunostaining of thin sections from the subgranular zone of the hippocampus showed that both groups of mice contained significantly fewer immature neurons (detected based on doublecortin expression), proliferating neural stem cells/precursor cells (detected based on BrdU labeling) and newborn neurons (detected based on simultaneous doublecortin staining and BrdU labeling) than unstressed controls (Figures 8C–F). The reductions were significantly more severe in animals showing stress-induced weight gain.

Animals with weight gain, but not those with weight loss, showed significantly less differentiation of neural stem cells/precursor cells into neurons than unstressed controls (Figure 8G). In the subgranular zone, animals showing weight gain contained significantly fewer new mature neurons (detected based on simultaneous NeuN staining and BrdU labeling) than animals showing weight loss (Figures 8H–J).

### 3.4 Stress-induced obesity in mice causes cognitive decline associated with inhibition of hippocampal neurogenesis and dysfunctional gut microbiota

**Figure 9:**
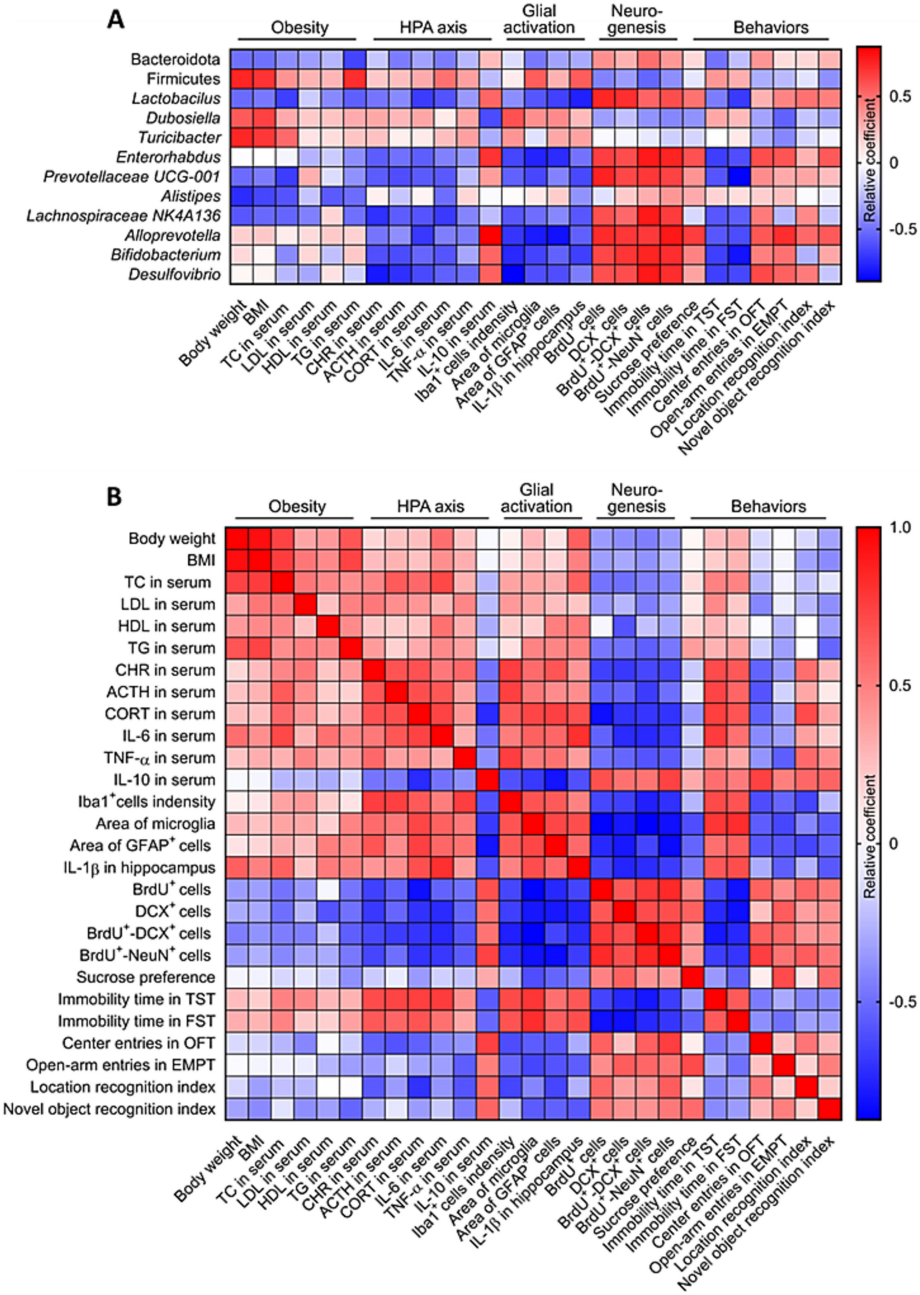
Correlation analysis of stress-induced obesity and gut microbiome, HPA axis, glial activation, neurogenesis and behaviors. **(A)** Correlation analysis between gut microbiome, stress-induced obesity, activation of HPA axis and hippocampal glia, impairment of hippocampal neurogenesis and cognitive decline. **(B)** Correlation analysis between obesity, gut microbiome, HPA axis, glial activation, neurogenesis and behaviors. The data used for correlation analysis were all derived from the control (Ctrl), weight gain (WG) and weight loss (WL) mice.

Given our observation that stress-induced obesity in mice causes hyperactivation of the HPA axis and hippocampal glia, inhibition of hippocampal neurogenesis and cognitive decline, we next wanted to know whether these changes are related to the disturbance of gut microbiome. Our results showed that the abundance of *Firmicutes*, *Dubosiella* and *Turicibacter* correlated positively with stress-induced obesity, activation of HPA axis and hippocampal glia, impairment of hippocampal neurogenesis and cognitive decline. The abundance of Bacteroidota, *Lactobacillus*, *Enterorhabdus*, *Prevotellaceae UCG-001*, *Alistipes*, *Lachnospiraceae NK4A136*, *Alloprevotella*, *Bifidobacterium* and *Desulfovibrio* correlated negatively with stress-induced obesity, activation of HPA axis and hippocampal glia, impairment of hippocampal neurogenesis and cognitive decline (Figure 9A). The stress-induced obesity correlated positively with hyperactivation of the HPA axis and hippocampal glia, impairment of hippocampal neurogenesis and cognitive decline. The hyperactivation of the HPA axis correlated positively with activation of hippocampal glia, and correlated negatively with hippocampal neurogenesis. The hyperactivation of hippocampal glia correlated negatively with hippocampal neurogenesis. The hippocampal neurogenesis correlated negatively with cognitive decline (Figure 9B). These results suggested that stress-induced obesity in mice causes cognitive decline associated with inhibition of hippocampal neurogenesis and dysfunctional gut microbiota.

**Figure 10:**
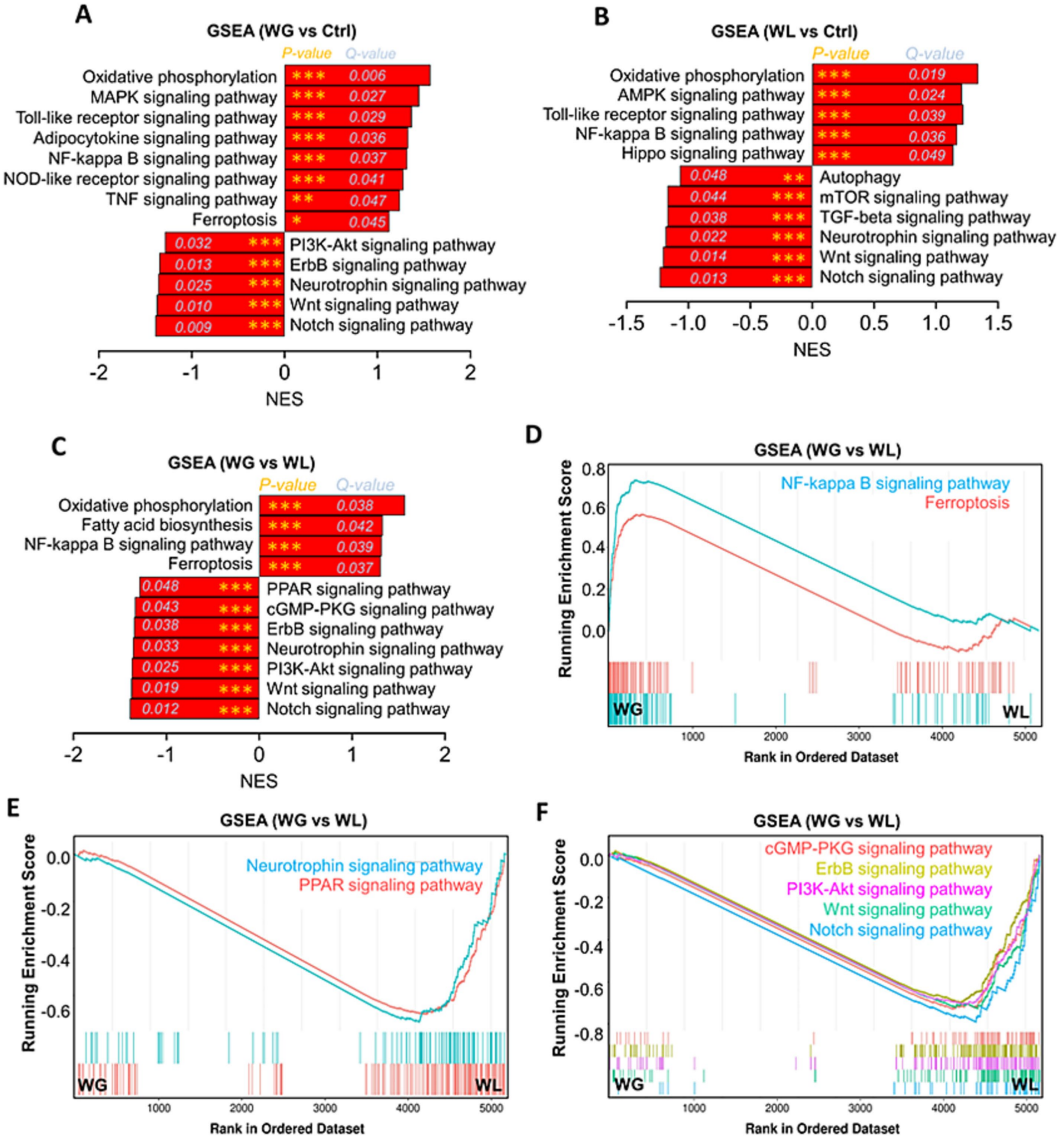
Gene set enrichment analysis of KEGG pathway based on differentially expressed genes among WG, WL and Ctrl mice. **(A)** Gene set enrichment analysis (GSEA) of Encyclopedia of Genes and Genomes (KEGG) pathway in the hippocampal transcriptome of weight gain (WG) mice compared to that of control (Ctrl) animals. **(B)** Gene set enrichment analysis of KEGG pathway in the hippocampal transcriptome of weight loss (WL) mice compared to that of Ctrl animals. **(C)** Gene set enrichment analysis of KEGG pathway in the hippocampal transcriptome of WG mice compared to that of WL animals after stress exposure. **(D–F)** Multichannel gene set enrichment analysis, indicating the differentially expressed genes that positively regulated NF-kappa B signaling pathway and ferroptosis, and the differentially expressed genes that negatively regulated PPAR signaling pathway, cGMP-PKG signaling pathway, ErbB signaling pathway, Neurotrophin signaling pathway, PI3K-Akt signaling pathway, Wnt signaling pathway and Notch signaling pathway were significantly enriched in hippocampus of WG mice compared to WL animals. Each line represents overlap between pairwise comparisons, based on gene set enrichment analysis.

To further explore the mechanism of overactivation of microglia and neurogenesis impairment in hippocampus of WG mice, gene set enrichment analysis of KEGG pathway based on differentially expressed genes among WG, WL and Ctrl mice were performed. Multichannel gene set enrichment analysis, indicating the differentially expressed genes that positively regulated oxidative phosphorylation, fatty acid biosynthesis, toll-like receptor signaling pathway, adipocytokine signaling pathway, NF-kappa B signaling pathway, NOD-like receptor signaling pathway, TNF signaling pathway and ferroptosis were significantly enriched in hippocampus of WG mice compared to WL or Ctrl animals (Figures 10A–D), suggesting that the hyperactivation of microglia and neuroinflammation in hippocampus induced by stress-mediated obesity are associated with the activation of these signaling pathways. In addition, we found that PPAR signaling pathway, a signaling pathway involved in lipid metabolism and anti-inflammation, was inhibited in hippocampus of WG mice compared to WL animals (Figure 10E). The signaling pathways (PI3K-AKT, cGMP-PKG, ErbB, neurotrophin, Wnt and Notch), which are involved in the proliferation, differentiation and survival of neural stem/precursor cells, were inhibited in hippocampus of WG mice compared to WL animals (Figures 10E,F), suggesting that the impairment in hippocampal neurogenesis of WG mice are associated with the activation of these signaling pathways.

## 4 Discussion

This study provides the first evidence that several pathologies previously reported in animals suffering stress-induced obesity while on a high-fat diet—hyperactivation of HPA axis and microglia, altered gut microbiome, and inhibited neurogenesis—also occur in animals suffering stress-induced obesity on a normal diet. In addition, we link these processes to cognitive deficit in the presence of stress-induced weight gain, but not in the presence of stress-induced weight loss.

Chronic mild stress led to significant weight loss in 50% of our animals, whereas 20% gained significantly in weight. These incidences are consistent with clinical observations that most patients with major depressive disorder lose weight (Kubera et al., 2022; Ma et al., 2019). The remaining 30% of mice did not suffer significant weight loss or gain during the stress paradigm, and these same animals showed no differences from unstressed controls in depression- or anxiety-like symptoms or in memory of objects or locations. In addition, these animals showed a significantly milder increase of corticosterone levels in serum than those that showed stress-induced obesity, even though the levels were still significantly higher than those in unstressed control animals. This group of animals may exhibit stress resistance (Zhang et al., 2021). We also found that WI mice showed a transient decrease in food consumption during the stress response period, while their food consumption returned to normal during the stress resistance period. The molecular basis of stress resistance should be studied further and may involve gene methylation (Dudek et al., 2021; Gonzalez et al., 2021) and life experiences before adulthood (Aydin et al., 2021; Liu et al., 2017; Rab and Admon, 2021).

While the combination of chronic stress and a high-fat diet is known to cause obesity (Liu et al., 2017; Slomp et al., 2019; Zhang et al., 2019), the molecules and pathways linking chronic mild stress to obesity in our mice on a normal diet remain unclear. Our experiments suggest several culprits. One is alterations in the gut microbiome. For example, stress-induced obesity in our animals was associated with a significant increase in the abundance of Firmicutes bacteria and significant decrease in the abundance of Bacteroidota bacteria. Firmicutes bacteria help individuals absorb food calories more effectively than Bacteroidota bacteria, which may increase risk of obesity (Baek et al., 2023; Da et al., 2020; Magne et al., 2020; Zhang et al., 2022).

A second culprit is HPA hyperactivation leading to release of corticotropin-releasing hormone, adrenocorticotropin and corticosterone, the levels of which were significantly elevated in mice showing stress-induced weight gain or loss, with the increases larger in those that gained weight. Stress-induced release of corticosterone from the adrenal cortex in rodents has been linked to obesity on a high-fat diet (Hueston and Deak, 2020; Kinlein et al., 2019; Lowrance et al., 2016; Qiu et al., 2019), at least in part because corticosterone hyperactivates glucocorticoid receptors in adipose tissue, leading to lipid accumulation (Appiakannan et al., 2020; Gado et al., 2023). At the same time, the hyperactivation of glucocorticoid receptors can cause inflammatory response (Bharti et al., 2019; Ferguson et al., 2020; Shimba and Ikuta, 2020; Wu et al., 2021), which may explain why levels of the pro-inflammatory cytokines tumor necrosis factor-*α* and interleukin-6 were significantly higher in the presence of stress-induced obesity than in its absence. Our results suggest that stress-induced hyperactivation of the HPA axis and peripheral inflammatory responses are more severe in the presence of obesity.

One pathway that explains our results is that stress hyperactivates the HPA axis and induces peripheral inflammation, which together alter the gut microbiome. In support of this idea, stress has been shown to alter the gut microbiota by promoting the secretion of neuroendocrine hormones such as corticotropin-releasing hormone, adrenocorticotropin and corticosterone (Guo et al., 2020; Liu et al., 2020), and changes in the gut microbiome have been associated with stress-induced obesity on a high-fat diet and cognitive changes (Wu et al., 2021; Chen et al., 2017; Rao et al., 2021; Xu et al., 2020). In other words, the gut-brain axis can bi-directionally regulate gut microbiota and brain response to stress (Deng et al., 2021; He et al., 2024; Westfall et al., 2021), which appears to be involved not only in stress-induced obesity but also in depression, anxiety, Alzheimer’s disease and irritable bowel syndrome (Liu et al., 2020; Westfall et al., 2021; Ma et al., 2022; Mosaferi et al., 2021). Indeed, our stressed mice showed significant depression and anxiety-like symptoms regardless of whether they gained or lost weight, though different symptoms predominated in the two groups. This may reflect differences in gut microbial composition between them. For example, the significantly lower abundance of *Lactobacillus* in the gut of animals showing stress-induced obesity may help explain their higher level of behavioral despair (Castaneda-Marquez et al., 2020; Wang et al., 2020). Future work should logitudinally analyze HPA activation, inflammatory responses, and the gut microbiome in order to identify the sequence in which they change under stress, which may clarify the mechanisms involved. Future experiments should also examine whether transplantation of fecal microbiota from animals with stress-induced obesity into naïve animals can reproduce the altered gene expression and activation of the HPA axis and hippocampal microglia that we observed here.

We detected microglial activation in the hippocampus of stressed animals regardless of whether they experienced significant weight gain or loss, with the activation more severe in the presence of weight gain. These results are consistent with the susceptibility of hippocampal microglia to corticosterone (Mao et al., 2020; Feng et al., 2019), metabolites of gut microbes (Chang et al., 2014), and inflammatory cytokines (Zhang et al., 2020). They are also consistent with studies implicating activation of hippocampal microglia in stress and depression (Jiang et al., 2022; Bassett et al., 2021; Zhang et al., 2023). Such activation has been linked to obesity on a high-fat diet (Kim et al., 2019). Our transcriptomic analysis suggests that stress-induced obesity on a normal diet causes dysfunction of hippocampal microglia through signaling mediated by NF-κB, Toll-like receptor and NOD-like receptor (Chen et al., 2022; Tang et al., 2019).

Whatever the processes mediating the hyperactivation of hippocampal microglia, this activation seems likely to cause the inhibition of neurogenesis observed in our stressed mice showing significant weight loss or gain, with the inhibition more severe in those showing weight gain. Hyperactivated microglia release tumor necrosis factor-*α* and interleukin-1β, which inhibit proliferation and differentiation of neural stem/precursor cells (Zhang et al., 2021; Liu et al., 2022). Our assay of interleukin-1β in serum in stressed animals is consistent with this. Inflammation may also contribute to the inhibition of neurogenesis (Zhang et al., 2020; Zhang et al., 2023). Our transcriptomic analysis suggests that microglial hyperactivation inhibits hippocampal neurogenesis by suppressing signaling mediated by PPARs, PI3K-Akt, Wnt and Notch, which normally promote the proliferation and differentiation of neural stem cells in hippocampus (Liu et al., 2022; Zhang et al., 2023). Whatever the pathways inhibiting hippocampal neurogenesis, our results link it to cognitive deficits that we observed in mice with stress-induced obesity, but not in mice with stress-induced weight loss. This is consistent with observations linking inhibition of neurogenesis to depression, anxiety and cognitive dysfunction (Li et al., 2023; Shen et al., 2022; Tunc-Ozcan et al., 2019; Zhang et al., 2023).

Our results should be interpreted with caution in light of several limitations. First, in present study, we screened only 20% of stress-induced obese animals from a small sample size, and this proportion needs to be confirmed in a larger sample size under non-high-fat diet conditions. Second, further work is needed to establish whether the gut microbiota is required for the obesity, and microglial, neurogenic and behavioral changes induced by stress exposure by fecal microbiota transplantation. In addition, the use of only male mice for this study, although narrowing the individual differences, also limits the applicability of the results of this study. Future work should replicate this experimental project in female mice, and explore how gut microbes affect proliferation and differentiation of adipose-derived stem cells. Despite these limitations, our work provides strong evidence that stress-induced obesity causes cognitive decline involving in gut microbiota disturbances and hippocampal neurogenesis impairment. These insights may provide treatment option for stress-induced obesity and cognitive decline by remodeling gut microbiota, inhibiting hyperactivation of hippocampal microglia and HPA axis.

## Data Availability

The datasets presented in this study can be found in online repositories. The names of the repository/repositories and accession number(s) can be found in the article/Supplementary material.

## Footnotes

^1^ https://cloud.majorbio.com
