# Supplementary material for: Stress-induced obesity in mice causes cognitive decline associated with inhibition of hippocampal neurogenesis and dysfunctional gut microbiota
Source: Front Microbiol. 2024 Oct 30;15:1381423. doi: 10.3389/fmicb.2024.1381423 (PMC11557545; doi:10.3389/fmicb.2024.1381423)
Supplement: Supplementary file 1 [file Table_1.docx]

**Supplementary Tables**

**Supplementary Table 1. The F value and P value in multiple comparisons of figure 1**

| **Marker** | | **Groups** | **Mean Diff.** | **Type of ANOVA** | **95.00% CI of diff.** | **F, DFn, DFd** | **P value** |
| --- | --- | --- | --- | --- | --- | --- | --- |
| *Changes in BMI* | *Row1* | WG vs. WL | 2.907 | Two-Way ANOVA  Tukey’s post hoc tests | -3.374 to 9.187 | \| F (2, 140) = 56.51 \| \| --- \| | 0.5180 |
|  |  | WG vs. WI | 0.8834 |  | -5.831 to 7.598 |  | 0.9479 |
|  |  | WL vs. WI | -2.023 |  | -7.331 to 3.285 |  | 0.6394 |
|  | *Row2* | WG vs. WL | -7.811 |  | -14.09 to -1.530 |  | 0.0105 |
|  |  | WG vs. WI | -14.56 |  | -21.28 to -7.850 |  | <0.0001 |
|  |  | WL vs. WI | -6.753 |  | -12.06 to -1.445 |  | 0.0086 |
|  | *Row3* | WG vs. WL | 18.05 |  | 11.77 to 24.33 |  | <0.0001 |
|  |  | WG vs. WI | 13.07 |  | 6.360 to 19.79 |  | <0.0001 |
|  |  | WL vs. WI | -4.979 |  | -10.29 to 0.3295 |  | 0.0710 |
|  | *Row4* | WG vs. WL | 20.78 |  | 14.49 to 27.06 |  | <0.0001 |
|  |  | WG vs. WI | 12.30 |  | 5.587 to 19.02 |  | <0.0001 |
|  |  | WL vs. WI | -8.474 |  | -13.78 to -3.166 |  | 0.0007 |
|  | *Row5* | WG vs. WL | 24.43 |  | 18.15 to 30.71 |  | <0.0001 |
|  |  | WG vs. WI | 11.03 |  | 4.313 to 17.74 |  | 0.0005 |
|  |  | WL vs. WI | -13.40 |  | -18.71 to -8.091 |  | <0.0001 |
| *Relative food consumption*  *(normalized to body weight)* | *Row1* | WG vs. WL | -2.705 | Two-Way ANOVA  Tukey’s post hoc tests | -10.59 to 5.181 | \| F (2, 135) = 4.104 \| \| --- \| | 0.6957 |
|  |  | WG vs. WI | 0.4424 |  | -8.161 to 9.046 |  | 0.9918 |
|  |  | WL vs. WI | 3.147 |  | -3.736 to 10.03 |  | 0.5259 |
|  | *Row2* | WG vs. WL | -1.805 |  | -9.690 to 6.080 |  | 0.8505 |
|  |  | WG vs. WI | 4.601 |  | -4.003 to 13.20 |  | 0.4161 |
|  |  | WL vs. WI | 6.406 |  | -0.4768 to 13.29 |  | 0.0738 |
|  | *Row3* | WG vs. WL | -0.2578 |  | -8.143 to 7.627 |  | 0.9967 |
|  |  | WG vs. WI | 4.624 |  | -3.979 to 13.23 |  | 0.4124 |
|  |  | WL vs. WI | 4.882 |  | -2.001 to 11.77 |  | 0.2162 |
|  | *Row4* | WG vs. WL | 3.053 |  | -4.832 to 10.94 |  | 0.6302 |
|  |  | WG vs. WI | 3.278 |  | -5.326 to 11.88 |  | 0.6394 |
|  |  | WL vs. WI | 0.2248 |  | -6.658 to 7.108 |  | 0.9967 |
|  | *Row5* | WG vs. WL | 0.3260 |  | -7.559 to 8.211 |  | 0.9947 |
|  |  | WG vs. WI | 3.607 |  | -4.996 to 12.21 |  | 0.5822 |
|  |  | WL vs. WI | 3.281 |  | -3.602 to 10.16 |  | 0.4975 |
| *TC in serum*  *(mmol/L)* | | Ctrl vs. WG | -2.216 | One-Way ANOVA  Tukey’s post hoc tests | -3.782 to -0.6498 | F (3, 16) = 9.599 | 0.0046 |
|  |  | Ctrl vs. WL | 0.5280 |  | -1.038 to 2.094 |  | 0.7710 |
|  |  | Ctrl vs. WI | -0.2460 |  | -1.812 to 1.320 |  | 0.9688 |
|  |  | WG vs. WL | 2.744 |  | 1.178 to 4.310 |  | 0.0007 |
|  |  | WG vs. WI | 1.970 |  | 0.4038 to 3.536 |  | 0.0116 |
|  |  | WL vs. WI | -0.7740 |  | -2.340 to 0.7922 |  | 0.5092 |
| *TG in serum*  *(mmol/L)* | | Ctrl vs. WG | -0.5120 | One-Way ANOVA  Tukey’s post hoc tests | -1.095 to 0.07135 | F (3, 16) = 4.423 | 0.0961 |
|  |  | Ctrl vs. WL | 0.1920 |  | -0.3913 to 0.7753 |  | 0.7833 |
|  |  | Ctrl vs. WI | 0.01400 |  | -0.5693 to 0.5973 |  | 0.9999 |
|  |  | WG vs. WL | 0.7040 |  | 0.1207 to 1.287 |  | 0.0156 |
|  |  | WG vs. WI | 0.5260 |  | -0.05735 to 1.109 |  | 0.0848 |
|  |  | WL vs. WI | -0.1780 |  | -0.7613 to 0.4053 |  | 0.8186 |
| *LDL in serum*  *(mmol/L)* | | Ctrl vs. WG | -0.3580 | One-Way ANOVA  Tukey’s post hoc tests | -1.021 to 0.3051 | F (3, 16) = 1.787 | 0.4358 |
|  |  | Ctrl vs. WL | 0.1380 |  | -0.5251 to 0.8011 |  | 0.9320 |
|  |  | Ctrl vs. WI | 0.05800 |  | -0.6051 to 0.7211 |  | 0.9943 |
|  |  | WG vs. WL | 0.4960 |  | -0.1671 to 1.159 |  | 0.1828 |
|  |  | WG vs. WI | 0.4160 |  | -0.2471 to 1.079 |  | 0.3114 |
|  |  | WL vs. WI | -0.08000 |  | -0.7431 to 0.5831 |  | 0.9853 |
| *HDL in serum*  *(mmol/L)* | | Ctrl vs. WG | 0.1200 | One-Way ANOVA  Tukey’s post hoc tests | -0.3052 to 0.5452 | F (3, 16) = 1.369 | 0.8500 |
|  |  | Ctrl vs. WL | -0.1500 |  | -0.5752 to 0.2752 |  | 0.7464 |
|  |  | Ctrl vs. WI | -0.1180 |  | -0.5432 to 0.3072 |  | 0.8561 |
|  |  | WG vs. WL | -0.2700 |  | -0.6952 to 0.1552 |  | 0.3018 |
|  |  | WG vs. WI | -0.2380 |  | -0.6632 to 0.1872 |  | 0.4055 |
|  |  | WL vs. WI | 0.03200 |  | -0.3932 to 0.4572 |  | 0.9963 |

**Table S2. The F value and P value in multiple comparisons of figure 2**

| **Marker** | | **Groups** | **Mean Diff.** | **Type of ANOVA** | **95.00% CI of diff.** | **F, DFn, DFd** | **P value** |
| --- | --- | --- | --- | --- | --- | --- | --- |
| *Coat score* | *Row1* | WG vs. WL | 0.1333 | Two-Way ANOVA  Tukey’s post hoc tests | -0.8226 to 1.089 | \| F (3, 195) = 52.80 \| \| --- \| | 0.9838 |
|  |  | WG vs. WI | -0.2222 |  | -1.265 to 0.8208 |  | 0.9459 |
|  |  | WG vs. Ctrl | 0.05128 |  | -0.9254 to 1.028 |  | 0.9991 |
|  |  | WL vs. WI | -0.3556 |  | -1.190 to 0.4788 |  | 0.6874 |
|  |  | WL vs. Ctrl | -0.08205 |  | -0.8319 to 0.6678 |  | 0.9920 |
|  |  | WI vs. Ctrl | 0.2735 |  | -0.5846 to 1.132 |  | 0.8421 |
|  | *Row2* | WG vs. WL | 0.1667 |  | -0.7892 to 1.123 |  | 0.9692 |
|  |  | WG vs. WI | -0.2778 |  | -1.321 to 0.7652 |  | 0.9008 |
|  |  | WG vs. Ctrl | -0.2949 |  | -1.272 to 0.6818 |  | 0.8624 |
|  |  | WL vs. WI | -0.4444 |  | -1.279 to 0.3899 |  | 0.5132 |
|  |  | WL vs. Ctrl | -0.4615 |  | -1.211 to 0.2883 |  | 0.3839 |
|  |  | WI vs. Ctrl | -0.01709 |  | -0.8752 to 0.8410 |  | >0.9999 |
|  | *Row3* | WG vs. WL | 0.2667 |  | -0.6892 to 1.223 |  | 0.8879 |
|  |  | WG vs. WI | -0.5556 |  | -1.599 to 0.4874 |  | 0.5132 |
|  |  | WG vs. Ctrl | -0.7949 |  | -1.772 to 0.1818 |  | 0.1539 |
|  |  | WL vs. WI | -0.8222 |  | -1.657 to 0.01216 |  | 0.0551 |
|  |  | WL vs. Ctrl | -1.062 |  | -1.811 to -0.3117 |  | 0.0018 |
|  |  | WI vs. Ctrl | -0.2393 |  | -1.097 to 0.6188 |  | 0.8880 |
|  | *Row4* | WG vs. WL | 0.8000 |  | -0.1559 to 1.756 |  | 0.1357 |
|  |  | WG vs. WI | -1.556 |  | -2.599 to -0.5126 |  | 0.0009 |
|  |  | WG vs. Ctrl | -1.795 |  | -2.772 to -0.8182 |  | <0.0001 |
|  |  | WL vs. WI | -2.356 |  | -3.190 to -1.521 |  | <0.0001 |
|  |  | WL vs. Ctrl | -2.595 |  | -3.345 to -1.845 |  | <0.0001 |
|  |  | WI vs. Ctrl | -0.2393 |  | -1.097 to 0.6188 |  | 0.8880 |
|  | *Row5* | WG vs. WL | 0.6000 |  | -0.3559 to 1.556 |  | 0.3661 |
|  |  | WG vs. WI | -2.000 |  | -3.043 to -0.9570 |  | <0.0001 |
|  |  | WG vs. Ctrl | -2.462 |  | -3.438 to -1.485 |  | <0.0001 |
|  |  | WL vs. WI | -2.600 |  | -3.434 to -1.766 |  | <0.0001 |
|  |  | WL vs. Ctrl | -3.062 |  | -3.811 to -2.312 |  | <0.0001 |
|  |  | WI vs. Ctrl | -0.4615 |  | -1.320 to 0.3966 |  | 0.5048 |
| *Sucrose preference* | *Row1* | WI vs. WL | 6.697 | Two-Way ANOVA  Tukey’s post hoc tests | -5.135 to 18.53 | \| F (3, 195) = 23.20 \| \| --- \| | 0.4596 |
|  |  | WI vs. WG | 5.438 |  | -7.472 to 18.35 |  | 0.6951 |
|  |  | WI vs. Ctrl | 1.270 |  | -10.82 to 13.36 |  | 0.9929 |
|  |  | WL vs. WG | -1.259 |  | -11.59 to 9.068 |  | 0.9890 |
|  |  | WL vs. Ctrl | -5.427 |  | -14.71 to 3.855 |  | 0.4304 |
|  |  | WG vs. Ctrl | -4.167 |  | -14.79 to 6.454 |  | 0.7399 |
|  | *Row2* | WI vs. WL | 6.700 |  | -5.131 to 18.53 |  | 0.4591 |
|  |  | WI vs. WG | 3.264 |  | -9.645 to 16.17 |  | 0.9136 |
|  |  | WI vs. Ctrl | -0.6842 |  | -12.77 to 11.40 |  | 0.9989 |
|  |  | WL vs. WG | -3.436 |  | -13.76 to 6.891 |  | 0.8243 |
|  |  | WL vs. Ctrl | -7.385 |  | -16.67 to 1.897 |  | 0.1695 |
|  |  | WG vs. Ctrl | -3.948 |  | -14.57 to 6.673 |  | 0.7705 |
|  | *Row3* | WI vs. WL | 4.734 |  | -7.098 to 16.57 |  | 0.7281 |
|  |  | WI vs. WG | 2.562 |  | -10.35 to 15.47 |  | 0.9556 |
|  |  | WI vs. Ctrl | -7.131 |  | -19.22 to 4.957 |  | 0.4224 |
|  |  | WL vs. WG | -2.172 |  | -12.50 to 8.155 |  | 0.9478 |
|  |  | WL vs. Ctrl | -11.87 |  | -21.15 to -2.584 |  | 0.0060 |
|  |  | WG vs. Ctrl | -9.693 |  | -20.31 to 0.9280 |  | 0.0874 |
|  | *Row4* | WI vs. WL | 8.909 |  | -2.922 to 20.74 |  | 0.2103 |
|  |  | WI vs. WG | -1.956 |  | -14.86 to 10.95 |  | 0.9794 |
|  |  | WI vs. Ctrl | -8.250 |  | -20.34 to 3.838 |  | 0.2917 |
|  |  | WL vs. WG | -10.86 |  | -21.19 to -0.5375 |  | 0.0350 |
|  |  | WL vs. Ctrl | -17.16 |  | -26.44 to -7.878 |  | <0.0001 |
|  |  | WG vs. Ctrl | -6.295 |  | -16.92 to 4.326 |  | 0.4182 |
|  | *Row5* | WI vs. WL | 13.43 |  | 1.595 to 25.26 |  | 0.0191 |
|  |  | WI vs. WG | -5.594 |  | -18.50 to 7.315 |  | 0.6759 |
|  |  | WI vs. Ctrl | -10.90 |  | -22.98 to 1.193 |  | 0.0936 |
|  |  | WL vs. WG | -19.02 |  | -29.35 to -8.694 |  | <0.0001 |
|  |  | WL vs. Ctrl | -24.32 |  | -33.60 to -15.04 |  | <0.0001 |
|  |  | WG vs. Ctrl | -5.302 |  | -15.92 to 5.319 |  | 0.5681 |
| *Sucrose consumption* | | Ctrl vs. WG | -11.14 | One-Way ANOVA  Tukey’s post hoc tests | -36.55 to 14.27 | F (3, 39) = 14.76 | 0.6450 |
|  |  | Ctrl vs. WL | 39.88 |  | 20.37 to 59.39 |  | <0.0001 |
|  |  | Ctrl vs. WI | 15.75 |  | -6.570 to 38.08 |  | 0.2474 |
|  |  | WG vs. WL | 51.02 |  | 26.15 to 75.89 |  | <0.0001 |
|  |  | WG vs. WI | 26.89 |  | -0.2395 to 54.03 |  | 0.0528 |
|  |  | WL vs. WI | -24.13 |  | -45.83 to -2.422 |  | 0.0243 |
| *Tail suspention test Latency (s)* | | Ctrl vs. WG | 22.62 | One-Way ANOVA  Tukey’s post hoc tests | -1.036 to 46.27 | F (3, 38) = 7.032 | 0.0653 |
|  |  | Ctrl vs. WL | 20.62 |  | 2.457 to 38.77 |  | 0.0208 |
|  |  | Ctrl vs. WI | -8.760 |  | -30.29 to 12.77 |  | 0.6960 |
|  |  | WG vs. WL | -2.000 |  | -25.15 to 21.15 |  | 0.9955 |
|  |  | WG vs. WI | -31.38 |  | -57.26 to -5.495 |  | 0.0122 |
|  |  | WL vs. WI | -29.38 |  | -50.35 to -8.396 |  | 0.0031 |
| *Tail suspention test Immobility time (s)* | | Ctrl vs. WG | -68.49 | One-Way ANOVA  Tukey’s post hoc tests | -105.4 to -31.60 | F (3, 42) = 12.02 | <0.0001 |
|  |  | Ctrl vs. WL | -30.76 |  | -59.08 to -2.434 |  | 0.0287 |
|  |  | Ctrl vs. WI | 4.759 |  | -25.16 to 34.68 |  | 0.9738 |
|  |  | WG vs. WL | 37.73 |  | 1.628 to 73.84 |  | 0.0375 |
|  |  | WG vs. WI | 73.25 |  | 35.88 to 110.6 |  | <0.0001 |
|  |  | WL vs. WI | 35.52 |  | 6.568 to 64.47 |  | 0.0108 |
| *Forced swimming test Latency (s)* | | Ctrl vs. WG | 16.13 | One-Way ANOVA  Tukey’s post hoc tests | 5.926 to 26.33 | F (3, 39) = 13.46 | 0.0007 |
|  |  | Ctrl vs. WL | 14.06 |  | 6.229 to 21.89 |  | 0.0001 |
|  |  | Ctrl vs. WI | -0.5385 |  | -9.502 to 8.425 |  | 0.9985 |
|  |  | WG vs. WL | -2.067 |  | -12.05 to 7.918 |  | 0.9445 |
|  |  | WG vs. WI | -16.67 |  | -27.56 to -5.773 |  | 0.0011 |
|  |  | WL vs. WI | -14.60 |  | -23.32 to -5.885 |  | 0.0003 |
| *Forced swimming test Immobility time (s)* | | Ctrl vs. WG | -82.97 | One-Way ANOVA  Tukey’s post hoc tests | -120.2 to -45.77 | F (3, 38) = 15.57 | <0.0001 |
|  |  | Ctrl vs. WL | -45.38 |  | -74.95 to -15.81 |  | 0.0011 |
|  |  | Ctrl vs. WI | -6.708 |  | -38.42 to 25.00 |  | 0.9409 |
|  |  | WG vs. WL | 37.59 |  | 0.3814 to 74.80 |  | 0.0469 |
|  |  | WG vs. WI | 76.27 |  | 37.34 to 115.2 |  | <0.0001 |
|  |  | WL vs. WI | 38.68 |  | 6.966 to 70.39 |  | 0.0116 |

**Table S3. The F value and P value in multiple comparisons of figure 3**

| **Marker** | | **Groups** | **Mean Diff.** | **Type of ANOVA** | **95.00% CI of diff.** | **F, DFn, DFd** | **P value** |
| --- | --- | --- | --- | --- | --- | --- | --- |
| *Training exploration time* | *FO* | Ctrl vs. WG | -3.260 | Two-Way ANOVA  Tukey’s post hoc tests | -15.19 to 8.671 | F (1, 80) = 8.832 | 0.8901 |
|  |  | Ctrl vs. WI | -4.123 |  | -15.16 to 6.913 |  | 0.7611 |
|  |  | Ctrl vs. WL | -0.9966 |  | -10.64 to 8.647 |  | 0.9930 |
|  |  | WG vs. WI | -0.8624 |  | -13.69 to 11.96 |  | 0.9980 |
|  |  | WG vs. WL | 2.263 |  | -9.386 to 13.91 |  | 0.9565 |
|  |  | WI vs. WL | 3.126 |  | -7.604 to 13.86 |  | 0.8702 |
|  | *NO* | Ctrl vs. WG | 3.260 |  | -8.671 to 15.19 |  | 0.8901 |
|  |  | Ctrl vs. WI | 4.123 |  | -6.913 to 15.16 |  | 0.7611 |
|  |  | Ctrl vs. WL | 0.9966 |  | -8.647 to 10.64 |  | 0.9930 |
|  |  | WG vs. WI | 0.8624 |  | -11.96 to 13.69 |  | 0.9980 |
|  |  | WG vs. WL | -2.263 |  | -13.91 to 9.386 |  | 0.9565 |
|  |  | WI vs. WL | -3.126 |  | -13.86 to 7.604 |  | 0.8702 |
| *Training location preference index* | | Ctrl vs. WG | -0.04826 | One-Way ANOVA  Tukey’s post hoc tests | -0.5348 to 0.4382 | F (3, 39) = 0.2545 | 0.9933 |
|  |  | Ctrl vs. WL | -0.04057 |  | -0.4141 to 0.3329 |  | 0.9912 |
|  |  | Ctrl vs. WI | -0.1377 |  | -0.5651 to 0.2897 |  | 0.8231 |
|  |  | WG vs. WL | 0.007696 |  | -0.4684 to 0.4838 |  | >0.9999 |
|  |  | WG vs. WI | -0.08942 |  | -0.6089 to 0.4301 |  | 0.9669 |
|  |  | WL vs. WI | -0.09712 |  | -0.5127 to 0.3185 |  | 0.9227 |
| *Object location test exploration time* | *FO* | Ctrl vs. WG | 12.40 | Two-Way ANOVA  Tukey’s post hoc tests | 0.3430 to 24.47 | F (1, 80) = 33.17 | 0.0414 |
|  |  | Ctrl vs. WI | -0.3807 |  | -11.54 to 10.78 |  | 0.9997 |
|  |  | Ctrl vs. WL | 7.844 |  | -1.905 to 17.59 |  | 0.1584 |
|  |  | WG vs. WI | -12.79 |  | -25.75 to 0.1806 |  | 0.0547 |
|  |  | WG vs. WL | -4.560 |  | -16.34 to 7.216 |  | 0.7406 |
|  |  | WI vs. WL | 8.225 |  | -2.623 to 19.07 |  | 0.2006 |
|  | *NO* | Ctrl vs. WG | -12.40 |  | -24.47 to -0.3430 |  | 0.0414 |
|  |  | Ctrl vs. WI | 0.3807 |  | -10.78 to 11.54 |  | 0.9997 |
|  |  | Ctrl vs. WL | -7.844 |  | -17.59 to 1.905 |  | 0.1584 |
|  |  | WG vs. WI | 12.79 |  | -0.1806 to 25.75 |  | 0.0547 |
|  |  | WG vs. WL | 4.560 |  | -7.216 to 16.34 |  | 0.7406 |
|  |  | WI vs. WL | -8.225 |  | -19.07 to 2.623 |  | 0.2006 |
| *Object location test location recognition index* |  | Ctrl vs. WG | 0.5596 | One-Way ANOVA  Tukey’s post hoc tests | 0.02166 to 1.098 | \| F (3, 39) = 4.432 \| \| --- \| \|  \| \|  \| | 0.0388 |
|  |  | Ctrl vs. WL | 0.3818 |  | -0.03121 to 0.7949 |  | 0.0789 |
|  |  | Ctrl vs. WI | -0.01217 |  | -0.4848 to 0.4605 |  | 0.9999 |
|  |  | WG vs. WL | -0.1778 |  | -0.7043 to 0.3487 |  | 0.8016 |
|  |  | WG vs. WI | -0.5718 |  | -1.146 to 0.002679 |  | 0.0515 |
|  |  | WL vs. WI | -0.3940 |  | -0.8536 to 0.06559 |  | 0.1154 |
| *Novel object recognition test exploration time* | *FO* | Ctrl vs. WG | -9.985 | Two-Way ANOVA  Tukey’s post hoc tests | -22.00 to 2.028 | F (1, 80) = 42.81 | 0.1374 |
|  |  | Ctrl vs. WI | -2.499 |  | -13.61 to 8.613 |  | 0.9348 |
|  |  | Ctrl vs. WL | -2.684 |  | -12.39 to 7.027 |  | 0.8868 |
|  |  | WG vs. WI | 7.487 |  | -5.427 to 20.40 |  | 0.4298 |
|  |  | WG vs. WL | 7.302 |  | -4.428 to 19.03 |  | 0.3660 |
|  |  | WI vs. WL | -0.1852 |  | -10.99 to 10.62 |  | >0.9999 |
|  | *NO* | Ctrl vs. WG | 9.985 |  | -2.028 to 22.00 |  | 0.1374 |
|  |  | Ctrl vs. WI | 2.499 |  | -8.613 to 13.61 |  | 0.9348 |
|  |  | Ctrl vs. WL | 4.395 |  | -5.316 to 14.11 |  | 0.6364 |
|  |  | WG vs. WI | -7.487 |  | -20.40 to 5.427 |  | 0.4298 |
|  |  | WG vs. WL | -5.590 |  | -17.32 to 6.139 |  | 0.5968 |
|  |  | WI vs. WL | 1.896 |  | -8.908 to 12.70 |  | 0.9674 |
| *Novel object recognition test location recognition index* |  | Ctrl vs. WG | 0.7948 | One-Way ANOVA  Tukey’s post hoc tests | 0.06128 to 1.528 | F (3, 40) = 2.829 | 0.0292 |
|  |  | Ctrl vs. WL | 0.2743 |  | -0.2806 to 0.8293 |  | 0.5528 |
|  |  | Ctrl vs. WI | 0.3015 |  | -0.3429 to 0.9460 |  | 0.5968 |
|  |  | WG vs. WL | -0.5205 |  | -1.232 to 0.1910 |  | 0.2199 |
|  |  | WG vs. WI | -0.4932 |  | -1.277 to 0.2901 |  | 0.3433 |
|  |  | WL vs. WI | 0.02723 |  | -0.5920 to 0.6465 |  | 0.9994 |

**Table S4. The F value and P value in multiple comparisons of figure 4**

| **Marker** | **Groups** | **Mean Diff.** | **Type of ANOVA** | **95.00% CI of diff.** | **F, DFn, DFd** | **P value** |
| --- | --- | --- | --- | --- | --- | --- |
| *ACTH in serum (pg/ml)* | Ctrl vs. WG | -60.00 | One-Way ANOVA  Tukey’s post hoc tests | -108.7 to -11.31 | F (3, 16) = 4.706 | 0.0134 |
|  | Ctrl vs. WL | -30.47 |  | -79.16 to 18.21 |  | 0.3133 |
|  | Ctrl vs. WI | -12.13 |  | -60.82 to 36.56 |  | 0.8905 |
|  | WG vs. WL | 29.53 |  | -19.16 to 78.21 |  | 0.3388 |
|  | WG vs. WI | 47.87 |  | -0.8158 to 96.56 |  | 0.0548 |
|  | WL vs. WI | 18.34 |  | -30.34 to 67.03 |  | 0.7074 |
| *CORT in serum (pg/ml)* | Ctrl vs. WG | -2.275 | One-Way ANOVA  Tukey’s post hoc tests | -3.311 to -1.238 | F (3, 16) = 16.04 | <0.0001 |
|  | Ctrl vs. WL | -1.210 |  | -2.247 to -0.1739 |  | 0.0195 |
|  | Ctrl vs. WI | -0.2929 |  | -1.329 to 0.7435 |  | 0.8495 |
|  | WG vs. WL | 1.064 |  | 0.02807 to 2.101 |  | 0.0431 |
|  | WG vs. WI | 1.982 |  | 0.9456 to 3.018 |  | 0.0003 |
|  | WL vs. WI | 0.9175 |  | -0.1189 to 1.954 |  | 0.0924 |
| *TNF-α in serum (pg/ml)* | Ctrl vs. WG | -11.64 | One-Way ANOVA  Tukey’s post hoc tests | -22.51 to -0.7631 | F (3, 16) = 3.582 | 0.0339 |
|  | Ctrl vs. WL | -5.272 |  | -16.14 to 5.600 |  | 0.5246 |
|  | Ctrl vs. WI | -2.037 |  | -12.91 to 8.835 |  | 0.9489 |
|  | WG vs. WL | 6.364 |  | -4.509 to 17.24 |  | 0.3682 |
|  | WG vs. WI | 9.599 |  | -1.274 to 20.47 |  | 0.0936 |
|  | WL vs. WI | 3.235 |  | -7.638 to 14.11 |  | 0.8293 |
| *IL-6 in serum (pg/ml)* | Ctrl vs. WG | -24.52 | One-Way ANOVA  Tukey’s post hoc tests | -40.95 to -8.099 | F (3, 16) = 7.411 | 0.0029 |
|  | Ctrl vs. WL | -6.736 |  | -23.16 to 9.690 |  | 0.6515 |
|  | Ctrl vs. WI | -2.645 |  | -19.07 to 13.78 |  | 0.9665 |
|  | WG vs. WL | 17.79 |  | 1.363 to 34.22 |  | 0.0315 |
|  | WG vs. WI | 21.88 |  | 5.454 to 38.31 |  | 0.0075 |
|  | WL vs. WI | 4.091 |  | -12.34 to 20.52 |  | 0.8907 |
| *IL-10 in serum (pg/ml)* | Ctrl vs. WG | 33.84 | One-Way ANOVA  Tukey’s post hoc tests | 14.12 to 53.55 | F (3, 16) = 9.623 | 0.0008 |
|  | Ctrl vs. WL | 29.70 |  | 9.985 to 49.42 |  | 0.0027 |
|  | Ctrl vs. WI | 18.85 |  | -0.8626 to 38.57 |  | 0.0634 |
|  | WG vs. WL | -4.136 |  | -23.85 to 15.58 |  | 0.9305 |
|  | WG vs. WI | -14.98 |  | -34.70 to 4.733 |  | 0.1727 |
|  | WL vs. WI | -10.85 |  | -30.56 to 8.868 |  | 0.4199 |

**Table S5. The F value and P value in multiple comparisons of figure 5**

| **Marker** | | **Groups** | **Mean Diff.** | **Type of ANOVA** | **95.00% CI of diff.** | **F, DFn, DFd** | **P value** |
| --- | --- | --- | --- | --- | --- | --- | --- |
| *Alpha diversity of fecal microbiota* | *Ace index* | Ctrl vs. WG | -88.72 | One-Way ANOVA  Tukey’s post hoc tests | -140.4 to -36.98 | F (2, 17) = 12.23 | 0.0011 |
|  |  | Ctrl vs. WL | -79.34 |  | -129.0 to -29.64 |  | 0.0021 |
|  |  | WG vs. WL | 9.376 |  | -42.36 to 61.11 |  | 0.8884 |
|  | *Chao index* | Ctrl vs. WG | -88.14 |  | -143.0 to -33.32 | F (2, 17) = 11.63 | 0.0019 |
|  |  | Ctrl vs. WL | -85.69 |  | -138.4 to -33.02 |  | 0.0017 |
|  |  | WG vs. WL | 2.452 |  | -52.37 to 57.28 |  | 0.9928 |
|  | *Shannon index* | Ctrl vs. WG | -0.1242 |  | -0.6497 to 0.4014 | F (2, 17) = 0.9141 | 0.8187 |
|  |  | Ctrl vs. WL | -0.2660 |  | -0.7709 to 0.2390 |  | 0.3875 |
|  |  | WG vs. WL | -0.1418 |  | -0.6673 to 0.3837 |  | 0.7712 |
|  | *Simpson index* | Ctrl vs. WG | -0.01486 |  | -0.05557 to 0.02584 | F (2, 17) = 0.7713 | 0.6253 |
|  |  | Ctrl vs. WL | 0.004047 |  | -0.03506 to 0.04315 |  | 0.9620 |
|  |  | WG vs. WL | 0.01891 |  | -0.02180 to 0.05961 |  | 0.4739 |
| *Relative abundances of phyla in fecal mirobiota* | *Bacteroidota* | Ctrl vs. WG | 13.59 | Two-Way ANOVA  Tukey’s post hoc tests | 3.277 to 23.91 | F (2, 51) = 180.9 | 0.0069 |
|  |  | Ctrl vs. WL | -1.500 |  | -11.41 to 8.411 |  | 0.9292 |
|  |  | WG vs. WL | -15.09 |  | -25.41 to -4.777 |  | 0.0025 |
|  | *Others* | Ctrl vs. WG | 6.053 |  | -4.262 to 16.37 |  | 0.3401 |
|  |  | Ctrl vs. WL | 8.020 |  | -1.891 to 17.93 |  | 0.1344 |
|  |  | WG vs. WL | 1.967 |  | -8.349 to 12.28 |  | 0.8901 |
|  | *Firmicutes* | Ctrl vs. WG | -19.65 |  | -29.96 to -9.330 |  | <0.0001 |
|  |  | Ctrl vs. WL | -6.520 |  | -16.43 to 3.391 |  | 0.2600 |
|  |  | WG vs. WL | 13.13 |  | 2.810 to 23.44 |  | 0.0094 |
| *Relative abundances of families in fecal mirobiota* | *Muribaculaceae* | Ctrl vs. WG | -1.683 | Two-Way ANOVA  Tukey’s post hoc tests | -11.90 to 8.536 | F (5, 102) = 89.80 | 0.9190 |
|  |  | Ctrl vs. WL | 4.314 |  | -5.504 to 14.13 |  | 0.5503 |
|  |  | WG vs. WL | 5.998 |  | -4.222 to 16.22 |  | 0.3469 |
|  | *Lactobacillaceae* | Ctrl vs. WG | 16.58 |  | 6.364 to 26.80 |  | 0.0006 |
|  |  | Ctrl vs. WL | 5.714 |  | -4.104 to 15.53 |  | 0.3529 |
|  |  | WG vs. WL | -10.87 |  | -21.09 to -0.6494 |  | 0.0343 |
|  | *Erysipelotrichaceae* | Ctrl vs. WG | -10.06 |  | -20.28 to 0.1601 |  | 0.0547 |
|  |  | Ctrl vs. WL | -0.9214 |  | -10.74 to 8.897 |  | 0.9729 |
|  |  | WG vs. WL | 9.138 |  | -1.082 to 19.36 |  | 0.0895 |
|  | *Lachnospiraceae* | Ctrl vs. WG | 1.255 |  | -8.965 to 11.47 |  | 0.9541 |
|  |  | Ctrl vs. WL | -0.6000 |  | -10.42 to 9.219 |  | 0.9884 |
|  |  | WG vs. WL | -1.855 |  | -12.07 to 8.365 |  | 0.9025 |
|  | *Prevotellaceae* | Ctrl vs. WG | -2.693 |  | -12.91 to 7.527 |  | 0.8058 |
|  |  | Ctrl vs. WL | -5.871 |  | -15.69 to 3.947 |  | 0.3333 |
|  |  | WG vs. WL | -3.179 |  | -13.40 to 7.041 |  | 0.7405 |
|  | *Others* | Ctrl vs. WG | -3.774 |  | -13.99 to 6.446 |  | 0.6552 |
|  |  | Ctrl vs. WL | -2.793 |  | -12.61 to 7.026 |  | 0.7777 |
|  |  | WG vs. WL | 0.9810 |  | -9.239 to 11.20 |  | 0.9717 |
| *Differences in relative abundance of individual genera* | *Lactobacilus* | Ctrl vs. WG | 18.52 | One-Way ANOVA  Tukey’s post hoc tests | 8.443 to 28.59 | F (2, 17) = 11.81 | 0.0006 |
|  |  | Ctrl vs. WL | 4.654 |  | -5.024 to 14.33 |  | 0.4503 |
|  |  | WG vs. WL | -13.86 |  | -23.93 to -3.788 |  | 0.0069 |
|  | *Dubosiella* | Ctrl vs. WG | -5.124 |  | -7.653 to -2.595 | F (2, 17) = 35.78 | 0.0002 |
|  |  | Ctrl vs. WL | 3.180 |  | 0.7500 to 5.610 |  | 0.0099 |
|  |  | WG vs. WL | 8.304 |  | 5.775 to 10.83 |  | <0.0001 |
|  | *Turicibacter* | Ctrl vs. WG | -5.048 |  | -7.665 to -2.431 | F (2, 17) = 17.11 | 0.0003 |
|  |  | Ctrl vs. WL | 0.3529 |  | -2.162 to 2.867 |  | 0.9313 |
|  |  | WG vs. WL | 5.401 |  | 2.784 to 8.018 |  | 0.0002 |
|  | *Enterorhabdus* | Ctrl vs. WG | -0.1379 |  | -1.743 to 1.467 | F (2, 17) = 15.00 | 0.9736 |
|  |  | Ctrl vs. WL | -2.949 |  | -4.490 to -1.407 |  | 0.0004 |
|  |  | WG vs. WL | -2.811 |  | -4.416 to -1.206 |  | 0.0009 |
|  | *Prevotellaceae UCG-001* | Ctrl vs. WG | -1.567 |  | -2.718 to -0.4163 | F (2, 17) = 13.80 | 0.0074 |
|  |  | Ctrl vs. WL | -2.206 |  | -3.311 to -1.100 |  | 0.0002 |
|  |  | WG vs. WL | -0.6388 |  | -1.790 to 0.5119 |  | 0.3512 |
|  | *Alistipes* | Ctrl vs. WG | 1.715 |  | 0.5973 to 2.833 | F (2, 17) = 8.161 | 0.0029 |
|  |  | Ctrl vs. WL | 0.4571 |  | -0.6169 to 1.531 |  | 0.5318 |
|  |  | WG vs. WL | -1.258 |  | -2.376 to -0.1401 |  | 0.0263 |
|  | *Lachnospiraceae NK4A136* | Ctrl vs. WG | -3.423 |  | -6.332 to -0.5144 | F (2, 17) = 7.697 | 0.0201 |
|  |  | Ctrl vs. WL | -3.974 |  | -6.769 to -1.180 |  | 0.0054 |
|  |  | WG vs. WL | -0.5512 |  | -3.460 to 2.358 |  | 0.8788 |
|  | *Alloprevotella* | Ctrl vs. WG | 1.352 |  | 0.8457 to 1.859 | F (2, 17) = 34.81 | <0.0001 |
|  |  | Ctrl vs. WL | 1.417 |  | 0.9305 to 1.904 |  | <0.0001 |
|  |  | WG vs. WL | 0.06500 |  | -0.4415 to 0.5715 |  | 0.9422 |
|  | *Bifidobacterium* | Ctrl vs. WG | 1.716 |  | 0.5445 to 2.888 | F (2, 17) = 8.989 | 0.0042 |
|  |  | Ctrl vs. WL | 1.549 |  | 0.4226 to 2.675 |  | 0.0069 |
|  |  | WG vs. WL | -0.1679 |  | -1.340 to 1.004 |  | 0.9286 |
|  | *Desulfovibrio* | Ctrl vs. WG | 1.198 |  | 0.5200 to 1.875 | F (2, 17) = 14.19 | 0.0008 |
|  |  | Ctrl vs. WL | 1.175 |  | 0.5241 to 1.826 |  | 0.0007 |
|  |  | WG vs. WL | -0.02247 |  | -0.7001 to 0.6552 |  | 0.9960 |

**Table S6. The F value and P value in multiple comparisons of figure 7**

| **Marker** | | **Groups** | **Mean Diff.** | **Type of ANOVA** | **95.00% CI of diff.** | **F, DFn, DFd** | **P value** |
| --- | --- | --- | --- | --- | --- | --- | --- |
| *Iba1^+^ number / mm^2^* | | Ctrl vs. WG | -137.9 | One-Way ANOVA  Tukey’s post hoc tests | -206.6 to -69.27 | F (2, 12) = 16.57 | 0.0005 |
|  |  | Ctrl vs. WL | -115.8 |  | -184.5 to -47.17 |  | 0.0019 |
|  |  | WG vs. WL | 22.10 |  | -46.57 to 90.77 |  | 0.6753 |
| *Quantification of the proportion of microglia hippocampus* | *Newborn Cells* | Ctrl vs. WG | 11.21 | Two-Way ANOVA  Tukey’s post hoc tests | 5.471 to 16.94 | F (2, 60) = 3.783e-030 | <0.0001 |
|  |  | Ctrl vs. WL | 7.532 |  | 1.795 to 13.27 |  | 0.0070 |
|  |  | WG vs. WL | -3.676 |  | -9.413 to 2.061 |  | 0.2798 |
|  | *Mature Cells* | Ctrl vs. WG | 20.55 |  | 14.81 to 26.28 |  | <0.0001 |
|  |  | Ctrl vs. WL | 13.03 |  | 7.291 to 18.76 |  | <0.0001 |
|  |  | WG vs. WL | -7.518 |  | -13.25 to -1.781 |  | 0.0071 |
|  | *Pre-activated Cells* | Ctrl vs. WG | -14.58 |  | -20.32 to -8.843 |  | <0.0001 |
|  |  | Ctrl vs. WL | -8.892 |  | -14.63 to -3.155 |  | 0.0012 |
|  |  | WG vs. WL | 5.688 |  | -0.04860 to 11.42 |  | 0.0524 |
|  | *Hyperactivated Cells* | Ctrl vs. WG | -16.22 |  | -21.96 to -10.49 |  | <0.0001 |
|  |  | Ctrl vs. WL | -7.132 |  | -12.87 to -1.395 |  | 0.0112 |
|  |  | WG vs. WL | 9.090 |  | 3.353 to 14.83 |  | 0.0010 |
|  | *Engulfed Cells* | Ctrl vs. WG | -0.9520 |  | -6.689 to 4.785 |  | 0.9162 |
|  |  | Ctrl vs. WL | -4.536 |  | -10.27 to 1.201 |  | 0.1474 |
|  |  | WG vs. WL | -3.584 |  | -9.321 to 2.153 |  | 0.2975 |
| *Area of microglia soma (μm2)* | | Ctrl vs. WG | -12.53 | One-Way ANOVA  Tukey’s post hoc tests | -17.54 to -7.523 | F (2, 12) = 22.45 | <0.0001 |
|  |  | Ctrl vs. WL | -7.215 |  | -12.22 to -2.206 |  | 0.0061 |
|  |  | WG vs. WL | 5.316 |  | 0.3080 to 10.32 |  | 0.0374 |
| *Length of branches (μm)* | | Ctrl vs. WG | 50.88 |  | 31.87 to 69.88 | F (2, 12) = 25.55 | <0.0001 |
|  |  | Ctrl vs. WL | 27.56 |  | 8.548 to 46.56 |  | 0.0059 |
|  |  | WG vs. WL | -23.32 |  | -42.33 to -4.312 |  | 0.0170 |
| *IL-1β level (pg/g)* | | Ctrl vs. WG | -21.61 |  | -31.25 to -11.96 | F (2, 12) = 17.88 | 0.0002 |
|  |  | Ctrl vs. WL | -10.04 |  | -19.68 to -0.3913 |  | 0.0413 |
|  |  | WG vs. WL | 11.57 |  | 1.922 to 21.21 |  | 0.0194 |
| *Area of GFAP+ staining (%)* | | Ctrl vs. WG | -4.765 |  | -6.892 to -2.638 | F (2, 12) = 18.89 | 0.0002 |
|  |  | Ctrl vs. WL | -3.374 |  | -5.501 to -1.247 |  | 0.0031 |
|  |  | WG vs. WL | 1.391 |  | -0.7360 to 3.518 |  | 0.2294 |

**Table S7. The F value and P value in multiple comparisons of figure 8**

| **Marker** | **Groups** | **Mean Diff.** | **Type of ANOVA** | **95.00% CI of diff.** | **F, DFn, DFd** | **P value** |
| --- | --- | --- | --- | --- | --- | --- |
| *DCX+ cells in per slice* | Ctrl vs. WG | 30.35 | One-Way ANOVA  Tukey’s post hoc tests | 15.30 to 45.40 | F (2, 12) = 14.51 | 0.0004 |
|  | Ctrl vs. WL | 16.47 |  | 1.425 to 31.52 |  | 0.0320 |
|  | Ctrl vs. WI | -13.88 |  | -28.92 to 1.171 |  | 0.0718 |
| *BrdU+ cells in per slice* | Ctrl vs. WG | 14.84 | One-Way ANOVA  Tukey’s post hoc tests | 8.709 to 20.98 | F (2, 12) = 20.85 | <0.0001 |
|  | Ctrl vs. WL | 7.152 |  | 1.017 to 13.29 |  | 0.0227 |
|  | Ctrl vs. WI | -7.692 |  | -13.83 to -1.557 |  | 0.0149 |
| *BrdU+-DCX+ cells in per slice* | Ctrl vs. WG | 12.76 | One-Way ANOVA  Tukey’s post hoc tests | 7.891 to 17.62 | F (2, 12) = 24.47 | <0.0001 |
|  | Ctrl vs. WL | 6.524 |  | 1.659 to 11.39 |  | 0.0098 |
|  | Ctrl vs. WI | -6.232 |  | -11.10 to -1.367 |  | 0.0131 |
| *BrdU+-DCX+ cells / BrdU+ cells (%)* | Ctrl vs. WG | 24.93 | One-Way ANOVA  Tukey’s post hoc tests | 7.352 to 42.51 | F (2, 12) = 7.226 | 0.0068 |
|  | Ctrl vs. WL | 10.37 |  | -7.207 to 27.95 |  | 0.2937 |
|  | Ctrl vs. WI | -14.56 |  | -32.14 to 3.018 |  | 0.1098 |
| *BrdU+-NeuN+ cells in per slice* | Ctrl vs. WG | 4.692 | One-Way ANOVA  Tukey’s post hoc tests | 2.804 to 6.580 | F (2, 12) = 22.26 | <0.0001 |
|  | Ctrl vs. WL | 2.802 |  | 0.9143 to 4.690 |  | 0.0050 |
|  | Ctrl vs. WI | -1.890 |  | -3.778 to -0.002336 |  | 0.0497 |
| *BrdU+-NenN+ cells / BrdU+ cells (%)* | Ctrl vs. WG | -2.695 | One-Way ANOVA  Tukey’s post hoc tests | -14.22 to 8.828 | F (2, 12) = 0.2940 | 0.8100 |
|  | Ctrl vs. WL | -3.015 |  | -14.54 to 8.508 |  | 0.7691 |
|  | Ctrl vs. WI | -0.3200 |  | -11.84 to 11.20 |  | 0.9970 |

**Table S8. The F value and P value in multiple comparisons of supplementary figure 1**

| **Marker** | | | **Groups** | **Mean Diff.** | **Type of ANOVA** | **95.00% CI of diff.** | **F, DFn, DFd** | **P value** |
| --- | --- | --- | --- | --- | --- | --- | --- | --- |
| *Time in open filed (s)* | | *Immobility* | Ctrl vs. WG | 4.714 | Two-Way ANOVA  Tukey’s post hoc tests | -14.89 to 24.32 | F (1, 84) = 2159 | 0.9220 |
|  |  |  | Ctrl vs. WI | 14.97 |  | -4.633 to 34.57 |  | 0.1956 |
|  |  |  | Ctrl vs. WL | -1.957 |  | -19.09 to 15.17 |  | 0.9906 |
|  |  |  | WG vs. WI | 10.26 |  | -11.05 to 31.57 |  | 0.5898 |
|  |  |  | WG vs. WL | -6.671 |  | -25.73 to 12.39 |  | 0.7956 |
|  |  |  | WI vs. WL | -16.93 |  | -35.99 to 2.133 |  | 0.0999 |
|  |  | *Movement* | Ctrl vs. WG | -4.697 |  | -24.30 to 14.90 |  | 0.9227 |
|  |  |  | Ctrl vs. WI | -14.95 |  | -34.56 to 4.649 |  | 0.1964 |
|  |  |  | Ctrl vs. WL | 1.976 |  | -15.15 to 19.11 |  | 0.9903 |
|  |  |  | WG vs. WI | -10.26 |  | -31.57 to 11.05 |  | 0.5898 |
|  |  |  | WG vs. WL | 6.673 |  | -12.39 to 25.73 |  | 0.7954 |
|  |  |  | WI vs. WL | 16.93 |  | -2.131 to 35.99 |  | 0.0998 |
| Time in open filed (s) | | *Immobility* | Ctrl vs. WG | 7.179 | Two-Way ANOVA  Tukey’s post hoc tests | -8.701 to 23.06 | F (1, 84) = 7847 | 0.6380 |
|  |  |  | Ctrl vs. WI | 3.312 |  | -12.57 to 19.19 |  | 0.9472 |
|  |  |  | Ctrl vs. WL | 7.003 |  | -6.874 to 20.88 |  | 0.5511 |
|  |  |  | WG vs. WI | -3.867 |  | -21.13 to 13.40 |  | 0.9357 |
|  |  |  | WG vs. WL | -0.1756 |  | -15.62 to 15.27 |  | >0.9999 |
|  |  |  | WI vs. WL | 3.691 |  | -11.75 to 19.13 |  | 0.9232 |
|  |  | *Movement* | Ctrl vs. WG | -7.256 |  | -23.14 to 8.623 |  | 0.6300 |
|  |  |  | Ctrl vs. WI | -3.790 |  | -19.67 to 12.09 |  | 0.9236 |
|  |  |  | Ctrl vs. WL | -7.030 |  | -20.91 to 6.847 |  | 0.5480 |
|  |  |  | WG vs. WI | 3.467 |  | -13.80 to 20.73 |  | 0.9525 |
|  |  |  | WG vs. WL | 0.2267 |  | -15.21 to 15.67 |  | >0.9999 |
|  |  |  | WI vs. WL | -3.240 |  | -18.68 to 12.20 |  | 0.9463 |
| *Open filed test center entries* | | | Ctrl vs. WG | 9.500 | One-Way ANOVA  Tukey’s post hoc tests | 3.074 to 15.93 | F (3, 39) = 7.038 | 0.0017 |
|  |  |  | Ctrl vs. WL | 6.800 |  | 1.866 to 11.73 |  | 0.0036 |
|  |  |  | Ctrl vs. WI | 5.556 |  | -0.09013 to 11.20 |  | 0.0552 |
|  |  |  | WG vs. WL | -2.700 |  | -8.989 to 3.589 |  | 0.6601 |
|  |  |  | WG vs. WI | -3.944 |  | -10.81 to 2.917 |  | 0.4227 |
|  |  |  | WL vs. WI | -1.244 |  | -6.734 to 4.245 |  | 0.9288 |
| *Time in elevated plus*  *maze (s)* | *Immobility* | | Ctrl vs. WG | 15.74 | Two-Way ANOVA  Tukey’s post hoc tests | -16.85 to 48.33 | F (1, 84) = 409.5 | 0.5868 |
|  |  |  | Ctrl vs. WI | 15.90 |  | -16.69 to 48.49 |  | 0.5790 |
|  |  |  | Ctrl vs. WL | 12.87 |  | -15.61 to 41.35 |  | 0.6380 |
|  |  |  | WG vs. WI | 0.1556 |  | -35.27 to 35.58 |  | >0.9999 |
|  |  |  | WG vs. WL | -2.869 |  | -34.56 to 28.82 |  | 0.9953 |
|  |  |  | WI vs. WL | -3.024 |  | -34.71 to 28.66 |  | 0.9945 |
|  | *Movement* | | Ctrl vs. WG | -15.79 |  | -48.38 to 16.80 |  | 0.5846 |
|  |  |  | Ctrl vs. WI | -15.93 |  | -48.52 to 16.66 |  | 0.5773 |
|  |  |  | Ctrl vs. WL | -12.89 |  | -41.37 to 15.59 |  | 0.6372 |
|  |  |  | WG vs. WI | -0.1444 |  | -35.57 to 35.28 |  | >0.9999 |
|  |  |  | WG vs. WL | 2.900 |  | -28.79 to 34.59 |  | 0.9951 |
|  |  |  | WI vs. WL | 3.044 |  | -28.64 to 34.73 |  | 0.9944 |
| *Time in elevated plus*  *maze (s)* | *Close-arm* | | Ctrl vs. WG | -32.16 | Two-Way ANOVA  Tukey’s post hoc tests | -51.99 to -12.33 | F (2, 126) = 1840 | 0.0003 |
|  |  |  | Ctrl vs. WI | -35.30 |  | -55.14 to -15.47 |  | <0.0001 |
|  |  |  | Ctrl vs. WL | -28.58 |  | -45.91 to -11.25 |  | 0.0002 |
|  |  |  | WG vs. WI | -3.144 |  | -24.71 to 18.42 |  | 0.9813 |
|  |  |  | WG vs. WL | 3.578 |  | -15.71 to 22.86 |  | 0.9627 |
|  |  |  | WI vs. WL | 6.722 |  | -12.56 to 26.01 |  | 0.8009 |
|  | *Center* | | Ctrl vs. WG | 4.477 |  | -15.36 to 24.31 |  | 0.9356 |
|  |  |  | Ctrl vs. WI | 5.099 |  | -14.73 to 24.93 |  | 0.9084 |
|  |  |  | Ctrl vs. WL | 3.024 |  | -14.31 to 20.36 |  | 0.9687 |
|  |  |  | WG vs. WI | 0.6222 |  | -20.94 to 22.18 |  | 0.9998 |
|  |  |  | WG vs. WL | -1.453 |  | -20.74 to 17.83 |  | 0.9973 |
|  |  |  | WI vs. WL | -2.076 |  | -21.36 to 17.21 |  | 0.9923 |
|  | *Open-arm* | | Ctrl vs. WG | 27.76 |  | 7.927 to 47.59 |  | 0.0022 |
|  |  |  | Ctrl vs. WI | 30.31 |  | 10.47 to 50.14 |  | 0.0007 |
|  |  |  | Ctrl vs. WL | 25.69 |  | 8.360 to 43.02 |  | 0.0010 |
|  |  |  | WG vs. WI | 2.544 |  | -19.02 to 24.11 |  | 0.9899 |
|  |  |  | WG vs. WL | -2.069 |  | -21.35 to 17.22 |  | 0.9924 |
|  |  |  | WI vs. WL | -4.613 |  | -23.90 to 14.67 |  | 0.9246 |
| *Traveled distances*  *in open field (m)* | | | Ctrl vs. WG | 1.877 | One-Way ANOVA  Tukey’s post hoc tests | -3.107 to 6.860 | F (3, 39) = 1.809 | 0.7443 |
|  |  |  | Ctrl vs. WL | -0.5246 |  | -4.351 to 3.302 |  | 0.9827 |
|  |  |  | Ctrl vs. WI | -2.601 |  | -6.980 to 1.777 |  | 0.3936 |
|  |  |  | WG vs. WL | -2.401 |  | -7.279 to 2.476 |  | 0.5554 |
|  |  |  | WG vs. WI | -4.478 |  | -9.800 to 0.8437 |  | 0.1258 |
|  |  |  | WL vs. WI | -2.077 |  | -6.334 to 2.181 |  | 0.5629 |
| *Open-arm entries* | | | Ctrl vs. WG | 8.756 | One-Way ANOVA  Tukey’s post hoc tests | 2.121 to 15.39 | F (3, 39) = 7.539 | 0.0056 |
|  |  |  | Ctrl vs. WL | 8.123 |  | 3.028 to 13.22 |  | 0.0007 |
|  |  |  | Ctrl vs. WI | 3.812 |  | -2.018 to 9.642 |  | 0.3103 |
|  |  |  | WG vs. WL | -0.6333 |  | -7.128 to 5.861 |  | 0.9936 |
|  |  |  | WG vs. WI | -4.944 |  | -12.03 to 2.142 |  | 0.2564 |
|  |  |  | WL vs. WI | -4.311 |  | -9.980 to 1.358 |  | 0.1909 |
| *Time in open-arms (%)* | | | Ctrl vs. WG | 9.835 | One-Way ANOVA  Tukey’s post hoc tests | 1.221 to 18.45 | F (3, 39) = 5.600 | 0.0198 |
|  |  |  | Ctrl vs. WL | 8.564 |  | 1.950 to 15.18 |  | 0.0067 |
|  |  |  | Ctrl vs. WI | 8.509 |  | 0.9413 to 16.08 |  | 0.0223 |
|  |  |  | WG vs. WL | -1.271 |  | -9.702 to 7.160 |  | 0.9773 |
|  |  |  | WG vs. WI | -1.326 |  | -10.52 to 7.873 |  | 0.9801 |
|  |  |  | WL vs. WI | -0.05445 |  | -7.413 to 7.304 |  | >0.9999 |
