# Supplementary material for: Stress-induced obesity in mice causes cognitive decline associated with inhibition of hippocampal neurogenesis and dysfunctional gut microbiota
Source: Front Microbiol. 2024 Oct 30;15:1381423. doi: 10.3389/fmicb.2024.1381423 (PMC11557545; doi:10.3389/fmicb.2024.1381423)
Supplement: Supplementary file 2 [file Data_Sheet_1.docx]

**Supplementary Figures**

**
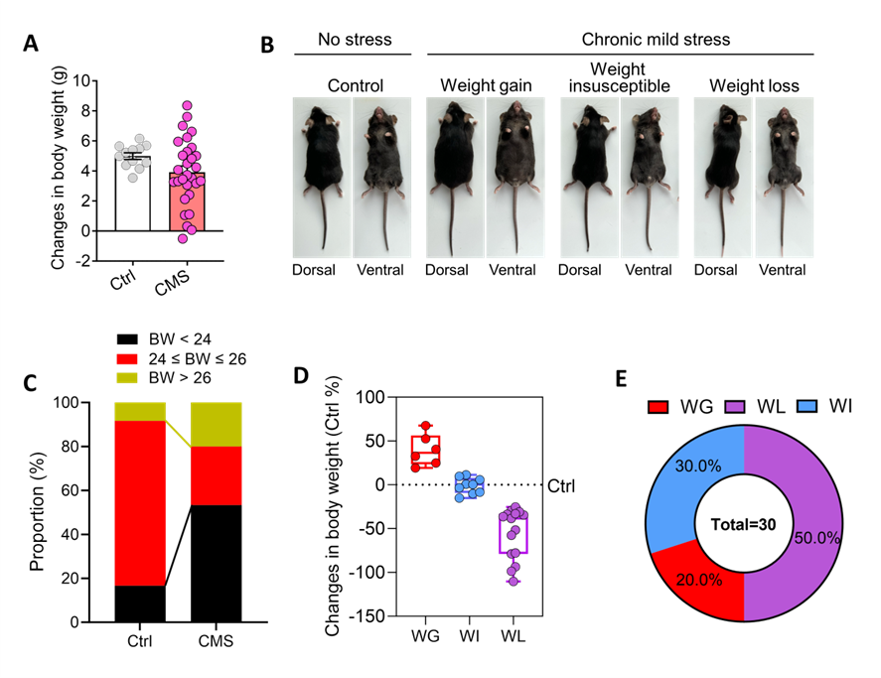
**

**Supplementary Figure 1. Differential effects of stress on body weight in mice**

**(A)** Change in body weight of mice after stress exposure.

**(B)** Photograph of the appearance of each representative mouse of control (Ctrl), weight gain (WG), weight insusceptible (WI) or weight loss (WL) mice.

**(C)** Change in the proportion of mice with different body weight after stress exposure.

**(D)** Change in body weight of WG, WI and WL mice relative to control mice.

**(E)** Quantification of the proportion of WG, WI and WL mice in stress-exposed mice.


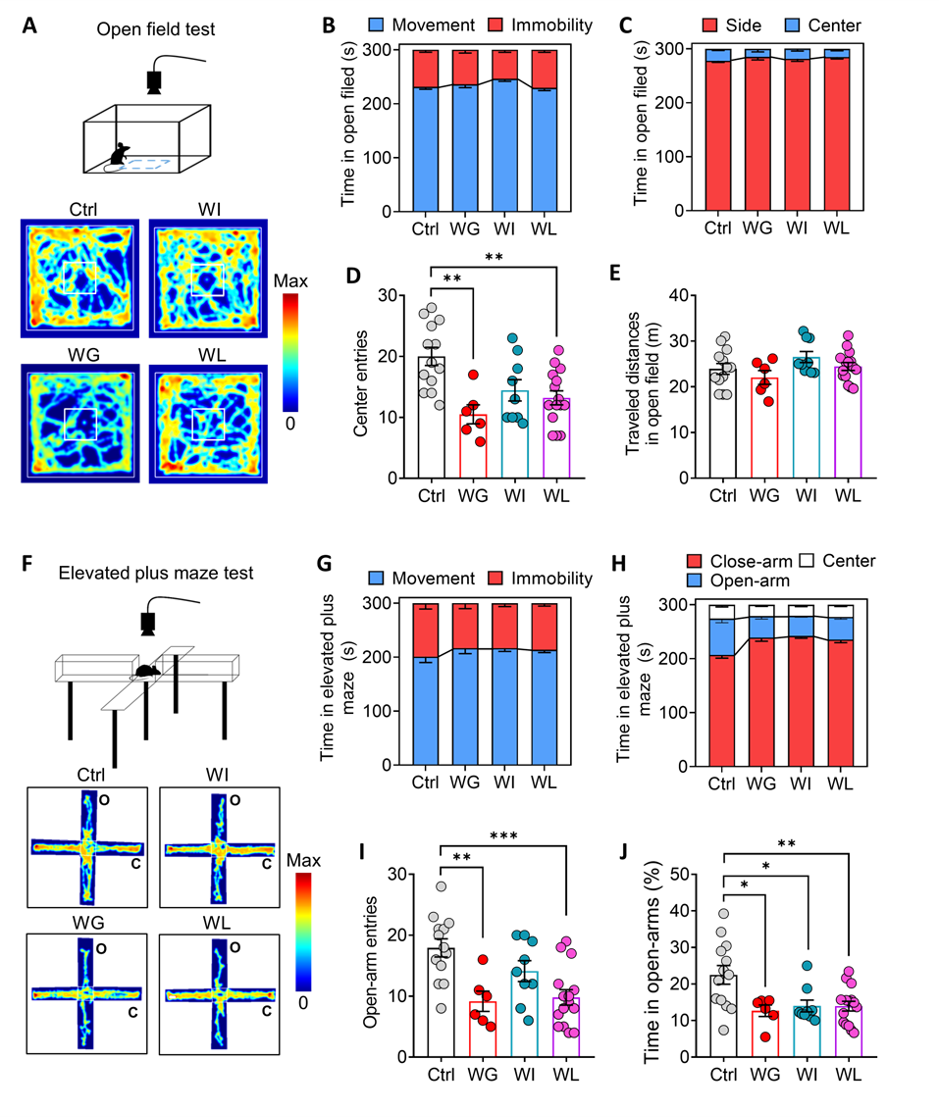


**Supplementary Figure 2. Differences between mice showing weight gain, insusceptible or loss after stress exposure in anxiety-like behaviors.**

**(A)** Heatmap of moving track from control (Ctrl), weight gain (WG), weight insusceptible (WI) or weight loss (WL) mice in the open field test. Ctrl, control animals never subjected to CMS.

**(B-E)** Quantization of (B) time spent in movement and immobility, (C) time spent in the center and side, (D) center entries, and (E) distance traveled by Ctrl, WG, WI or WL mice.

**(F)** Heatmap of mouse behavior in the elevated plus maze. “O” means open-arm, and “C” means close-arm.

**(G-J)** Quantization of (G) time spent in movement and immobility, (H) time spent in the open-arm, close-arm and center, (I) open-arm entries, and (J) time spent in open-arm by Ctrl, WG, WI or WL mice. Ctrl, control animals never subjected to CMS.

Data are mean ± standard error of the mean (SEM). Quantitative results come from 6 – 14 animals per condition. **P* < 0.05; ***P* < 0.01; ****P* < 0.001, based on one-way ANOVA followed by Tukey's multiple-comparisons test.


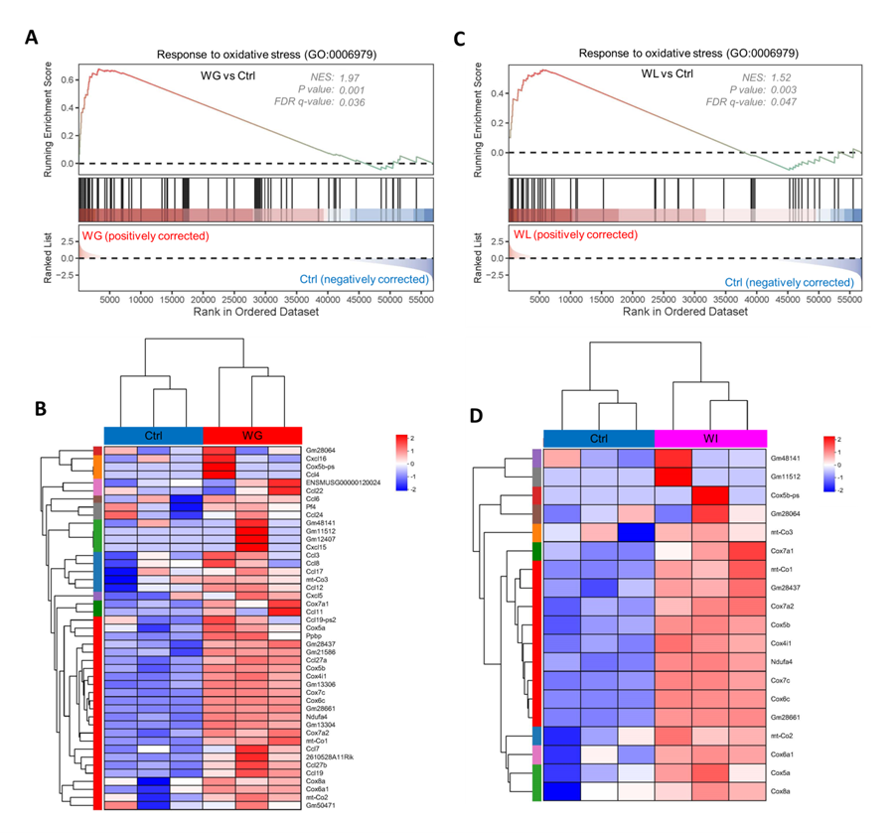


**Supplementary Figure 3. Gene set enrichment analysis of gene ontology (GO) terms based on differentially expressed genes between WG and Ctrl mice, or between WL and Ctrl mice.**

**(A)** Gene set enrichment analysis, indicating the differentially expressed genes that positively regulated “response to oxidative stress (GO:0006979)” were significantly enriched in hippocampus of WG mice compared to Ctrl animals. Each line represents overlap between pairwise comparisons, based on gene set enrichment analysis.

**(B)** Hierarchical cluster analysis of enriched differentially expressed genes related to response to oxidative stress between WG and Ctrl.

**(C)** Gene set enrichment analysis, indicating the differentially expressed genes that positively regulated “response to oxidative stress (GO:0006979)” were significantly enriched in hippocampus of WL mice compared to Ctrl animals. Each line represents overlap between pairwise comparisons, based on gene set enrichment analysis.

**(D)** Hierarchical cluster analysis of enriched differentially expressed genes related to response to oxidative stress between WL and Ctrl.


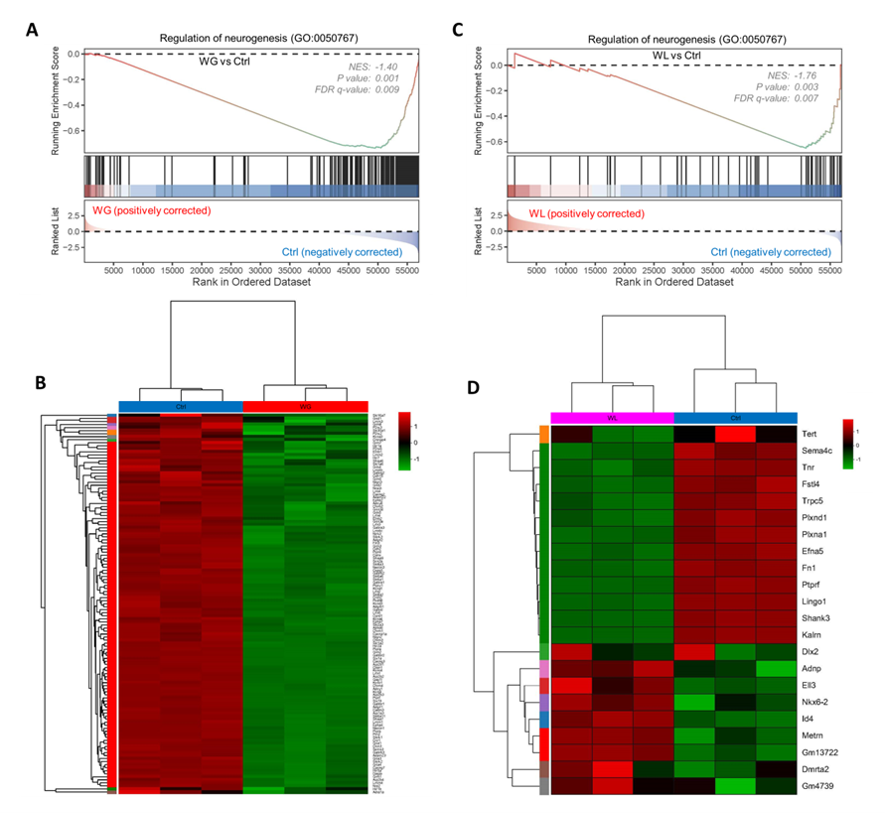


**Supplementary Figure 4. Mice that exhibited stress obesity were accompanied by exaggerated impairment in hippocampal neurogenesis.**

**(A)** Gene set enrichment analysis, indicating the differentially expressed genes that negatively regulated “regulation of neurogenesis (GO:0050767)” were significantly enriched in hippocampus of WG mice compared to Ctrl animals. Each line represents overlap between pairwise comparisons, based on gene set enrichment analysis.

**(B)** Hierarchical cluster analysis of enriched differentially expressed genes related to response to oxidative stress between WG and Ctrl.

**(C)** Gene set enrichment analysis, indicating the differentially expressed genes that negatively regulated “regulation of neurogenesis (GO:0050767)” were significantly enriched in hippocampus of WL mice compared to Ctrl animals. Each line represents overlap between pairwise comparisons, based on gene set enrichment analysis.

**(D)** Hierarchical cluster analysis of enriched differentially expressed genes related to response to oxidative stress between WL and Ctrl.
